# Characterization of *Triticum aestivum* Abscisic Acid Receptors and a Possible Role for These in Mediating Fusairum Head Blight Susceptibility in Wheat

**DOI:** 10.1371/journal.pone.0164996

**Published:** 2016-10-18

**Authors:** Cameron S. Gordon, Nandhakishore Rajagopalan, Eddy P. Risseeuw, Marci Surpin, Fraser J. Ball, Carla J. Barber, Leann M. Buhrow, Shawn M. Clark, Jonathan E. Page, Chris D. Todd, Suzanne R. Abrams, Michele C. Loewen

**Affiliations:** 1 Department of Biochemistry, University of Saskatchewan, 107 Wiggins Rd., Saskatoon, SK, S7N 5E5, Canada; 2 National Research Council of Canada, 110 Gymnasium Place, Saskatoon, SK, S7N 0W9, Canada; 3 Valent BioSciences Corporation, 870 Technology Way, Libertyville, Illinois, 60048, United States of America; 4 Department of Biology, University of Saskatchewan, 112 Science Place, Saskatoon, SK, S7N 5E2, Canada; 5 Department of Chemistry, University of Saskatchewan, 110 Science Place, Saskatoon, SK, S7N 5C9, Canada; Murdoch University, AUSTRALIA

## Abstract

Abscisic acid (ABA) is a well-characterized plant hormone, known to mediate developmental aspects as well as both abiotic and biotic stress responses. Notably, the exogenous application of ABA has recently been shown to increase susceptibility to the fungal pathogen *Fusarium graminearum*, the causative agent of *Fusarium* head blight (FHB) in wheat and other cereals. However roles and mechanisms associated with ABA’s modulation of pathogen responses remain enigmatic. Here the identification of putative ABA receptors from available genomic databases for *Triticum aestivum* (bread wheat) and *Brachypodium distachyon* (a model cereal) are reported. A number of these were cloned for recombinant expression and their functionality as ABA receptors confirmed by *in vitro* assays against protein phosphatases Type 2Cs. Ligand selectivity profiling of one of the wheat receptors (Ta_PYL2DS_FL) highlighted unique activities compared to *Arabidopsis* AtPYL5. Mutagenic analysis showed Ta_PYL2DS_FL amino acid D180 as being a critical contributor to this selectivity. Subsequently, a virus induced gene silencing (VIGS) approach was used to knockdown wheat Ta_PYL4AS_A (and similar) *in planta*, yielding plants with increased early stage resistance to FHB progression and decreased mycotoxin accumulation. Together these results confirm the existence of a family of ABA receptors in wheat and *Brachypodium* and present insight into factors modulating receptor function at the molecular level. That knockdown of Ta_PYL4AS_A (and similar) leads to early stage FHB resistance highlights novel targets for investigation in the future development of disease resistant crops.

## Introduction

Abscisic acid (ABA) is a phytohormone that plays well-established roles in plant development (seed dormancy, germination, seedling growth and seed deposition) and abiotic stress (drought, high salt, high temperature) responses [1]. The last decade has also seen an eruption of new knowledge addressing the mechanisms by which ABA responses are elicited at the molecular level. One of the most significant findings was the identification of a family of soluble ABA receptors [2, 3]. These are small START (steroidogenic acute regulatory-related lipid transfer) domain proteins comprising a portion of the larger Betv1 superfamily. Thirteen functional ABA receptor homologs were originally identified and characterized in *Arabidopsis thaliana*, and entitled Regulatory Component of ABA Receptors (RCARs) or Pyrabactin Resistant/Pyrabactin Resistant-like (PYR/PYL) receptors. Structure-function studies have shown that ABA binding in a cavity in the receptor leads to a gate-latch conformational closure of the pocket, yielding a surface on the receptor that has high affinity for the catalytic site on Protein Phosphatase 2C proteins (PP2Cs) [4–8]. Binding of the ABA-stimulated receptor to the PP2C blocks substrate access, yielding a non-competitive inhibition of the phosphatase, releasing the downstream positive transcriptional effectors Sucrose-Non fermenting Kinase1-related protein kinases (e.g. SnRK2; reviewed in [9, 10]).

Equally expansive families of ABA receptors have since been identified in a variety of dicot species including strawberry [11–13], grape [14, 15], citrus [16], cucumber [17] and soy bean [18], with roles for these receptors broadly correlated to ABA sensitivity, ripening and stress perception processes. On the side of monocots, both the rice and barley ABA receptor families have been investigated in some detail, with select receptors shown to mediate the usual developmental and abiotic responses *in planta* [19–22].

Aside from its well-documented roles in development and abiotic stress responses, ABA has also been implicated more recently in modulation of responses to various diseases of plants. Although ABA can impact resistance either positively or negatively depending on the pathogen, the tide of evidence is leaning more toward ABA working as a susceptibility factor, at least with respect to fungal pathogens [23–26]. On the side of bacterial response, AtRCAR3 has been shown to increase resistance to *Pseudomonas syringae*, through modulation of Arabidopsis stomatal aperture [27]. In the case of fungal responses, ABA has been shown to increase the susceptibility of *Arabidopsis* to *Fusarium oxysporum* [28, 29], while knockdown of ABA signalling machinery was shown to increase resistance to *Plectosphaerella cucumerina* [30]. In the case of cereal crops, spraying ABA on rice plants increased the severity of rice blast fungus *M*. *grisea* infection [31], while more recently, exogenous application of ABA was shown to increase development of disease symptoms of *M*. *Oryzae* on barley [32].

Furthermore on the monocot side, two recent reports show that priming wheat heads with ABA leads to increased susceptibility to *Fusarium graminearum*, the causative agent of *Fusarium* head blight (FHB) [33, 34]. FHB is fungal disease of wheat and other small grains that leads to shriveled and/or discolored grain contaminated with the mycotoxin dioxynivalonol (DON) [35–37]. The recent priming studies showed that the effect of exogenous ABA was not linked to modulation of pathogen growth or sporulation in axenic conditions, trichothecene gene expression, deoxynivalenol accumulation, or salicylic acid (SA) / jasmonic acid (JA) accumulation, but might be linked to promotion of the expression of pathogen early-infection genes including hydrolases and cytoskeletal reorganization genes [34]. At the same time, *F*. *graminearum* was shown to produce ABA itself, emphasizing a naturally occurring connection between this hormone and the pathogen [33]. In many instances the effects of ABA are proposed to be linked to antagonistic interactions between components of the ABA signaling pathway and those of the classical defense response hormones including salicylic acid, jasmonic acid and ethylene (reviewed in [26, 33]).

Herein a bioinformatics search of available draft genomic sequence that identified putative ABA receptors in wheat (*Triticum aestivum* c.v. ‘Chinese Spring’), as well as the model monocot *Brachypodium distachyon* is described. A number of these were validated as ABA receptors *in vitro*. Further comparative analyses using chemical analogs as ligands highlighted unique ABA-analog selectivities for one of the wheat receptors compared to a close *A*. *thaliana*ortholog. This selectivity was linked to variability at amino acid residue 180, situated at the receptor-PP2C interface, highlighting the importance of this interaction in receptor functionality. Finally knockdown of wheat ABA receptor(s) was shown to increase resistance to the fungal pathogen *F*. *graminearum* and reduced accumulation of the mycotoxin DON in grain. Overall this work highlights the existence of ABA receptors families in wheat and the model monocot *B*. *distachyon*, members of which may function as positive effectors of FHB infection in wheat.

## Methods

### Chemicals

All chemicals and reagents were purchased from Sigma-Aldrich unless otherwise indicated. ABA and ABA-analogs were synthesized as previously described [38].

### Bioinformatics

Putative ABA receptors were identified by using the National Center for Biotechnology Information (NCBI) blastn or tblastn [39] or the *B*. *distachion* genomic database (www.plantgdb.org/BdGDB/) using the *Arabidopsis* AtPYL5 (accession NP_196163) cDNA sequence. In the case of *T*. *aestivum*, initially the *Arabidopsis* AtPYL5 cDNA sequence was also used as a blast query against all available wheat sequences in the NCBI database where one hit (accession AK335719) was subsequently used as a template to search the wheat (*T*. *aestivum* cv ‘Chinese Spring’), International Wheat Genomics Sequencing Consortium (IWGSC) survey sequence and Wheat reference sequence chromosome 3B databases by blastn and tblastn (https://urgi.versailles.inra.fr/blast/). Blast hits were aligned using Clustal Omega [40] and their evolutionary relationship investigated using Mega6 [41].

### Recombinant Protein Expression and Enrichment

A cDNA encoding a codon-optimized putative ABA receptor Ta_PYL2DS_FL (Table 1) as well as the 7 variants of this sequence and a non-codon optimized wheat PP2C phosphatase TaABI1 (accession AB238930; [42]; Table 1) were synthesized and cloned into expression vector pEX-N-His for recombinant expression of N-terminally His tagged proteins in *Escherchia coli* (Blue Heron DNA Synthesis, USA). These were individually transformed in BL21-Rosetta (DE3) competent cells and grown in on Luria Broth (LB) containing 100 μg/mL ampicillin and incubated overnight at 37°C with shaking. This starter culture was used to inoculate (1:50) fresh cultures, which were incubated at 37°C with shaking until and OD600 of 0.4–0.6 was obtained. Protein expression was induced with the addition of 1 mM IPTG and were further grown for 12 hours at 16°C with shaking. Cells were harvested by centrifugation at 3283 x g for 15 min at 4°C and pellets stored frozen at -80°C. Expression of *A*. *thaliana* ABA receptors and PP2Cs was a carried out similarly, and exactly as described previously [38]. Recombinant protein was enriched by Ni-NTA affinity chromatography. Cell pellets from large 1.5 L induction cultures were re-suspended in 20–30 mL of protein purification Buffer A (100 mM tris-HCl (pH 7.9), 100 mM NaCl, 0.3 mM MnCl2, 4 mM DTT) containing 10 mM imidazole. Resuspended cells were lysed by French Press on ice and the cell lysates was centrifuged at 39,800 x g for 30 minutes at 4°C. Ni-NTA resin (5 ml; Qiagen, USA) was washed three times with 5 mL of lysis buffer containing 10 mM imidazole. The supernatant of the cell lysate incubated with the resin at 4°C for 45 minutes with gentle shaking to facilitate protein binding to the resin. The flow through was collected, and the column was washed three times with 5 mL lysis buffer containing 20 mM imidazole and collected. Proteins were eluted with lysis buffer containing 150 mM imidazole (two 5 mL elutions). Protein concentrations were determined using the Bio-Rad Protein Assay kit following manufacturer’s instructions (Bio-Rad; [43]).

**Table 1:** *T*. *aestivum* and *B*. *distachyon* putative ABA receptor genes.

Name (used in this paper)	Gene Locator ID*	% (predicted) amino acid identity to AtPYL5	Sequence Length (amino acids)
AtPYL5 (AT5g05440)	**NM_120626.2**	100	203
Bradi2g32250	XP_003568737	42	210
Bradi3g09580	XP_003572108	44	210
Bradi1g37810	XP_003563747	64	196
Bradi3g08580	XP_003571767	55	239
Bradi2g22510	XP_003566151	52	197
Bradi2g53840	XP_003567239	44	213
Bradi1g16710	XP_003559749	49	213
Bradi1g65130	XP_003558125	49	227
Bradi3g34070	XP_003572115	50	221
Ta_1AL	1AL_3980541:1795..2273;1AL_459150:1731..1576	52	204
Ta_1AS	1AS_3273118:1331..1134	55	66
Ta_1BS	1BS_3442744:3415..3218;1BS_2995372:2..226	56	125
Ta_1DS	1DS_1883027:3646..3449	55	66
Ta_2BS_A	2BS_996029:2..211	62	70
Ta_2BS_B	2BS_2443581:836..615	70	74
**Ta_2DS** **Ta_2DS_FL**	2DS_5389215:2..238 AK335719	68 59	79 214
Ta_3AS	3AS_3337229:1212..727	56	162
Ta_3B_A	3B_10567320:9764..9177	59	196
Ta_3B_B	3B_10745450:1307..822	56	162
Ta_3DS	3DS_2593515:931..749	57	61
Ta_4AS_A	4AS_5927184:7130..7657	62	176
Ta_4AS_B	4AS_5976031:1..285	58	95
Ta_4BL	4BL_6970657:9524..10051	62	176
Ta_4DL	4DL_14307801:11405..11932	62	176
Ta_6AL	6AL_5708018:13522..13400	61	41
Ta_6DL	6DL_873316:1..96	59	32
Ta_7AL	7AL_4517258:8263..8069	47	65
Ta_7BL	7BL_6668505:3035..2790	47	82
Ta_7DL	7DL_2074074:1723..1529	47	65
Ta_CDM82373	CDM82373	53	201

* Gene Locators are NCBI Gene Identifiers whenever available. For wheat sequences where GI numbers are not available the identifier refers to the IWGSC sequence survey database, for contigs longer than 200 for each chromosome.

### ABA Receptor Assay

Receptors were tested for activity as previously described [38]. Protein encoding receptors and PP2Cs were mixed at an approximate molar ratio of 10:1 receptor (0.024 μg/μL final concentration) to phosphatase (0.004 μg/μL final concentration), with ABA or ABA analog (0.1 μM unless otherwise noted) and buffer containing 100 mM Tris pH 7.9, 100 mM NaCl, 0.3 mM MnCl2 and 4 mM DTT in a total final volume of 50 μL. The mixture was incubated for 15 min at 30°C. Fifty μL of substrate (1 mM 4-Methylumbelliferyl phosphate) was then added to initiate the reaction and the assay mixture was incubated at 30°C. The intensity of the fluorescent product was the measured using a Perkin Elmer Victor 3 V 1420 fluorescent plate reader at 30 min after initiation of the assay. The excitation wavelength was 355 nm, the emission wavelength was 460 nm, and the measurement time was 0.1 s.

### Plant Material

The cultivar ‘Fielder’ was used for isolation of cDNA in all wheat experiments. Normal growing conditions were 16/8 hour photoperiod, full light; 25°C day/20°C nightin Sunshine mix #8 (Sun Gro). Plants were given 20-20-20 NPK fertilizer mix once a week. Rhapsody, Seranade and Intercept (Bayer Crop Science) were applied when necessary during plant development for pathogen and pest management. *Nicotiana benthamiana* plants were grown under 16/8 hour photoperiod, full light; 25°C day/20°C night in Sunshine mix #8 (Sun Gro) supplemented with slow release fertilizer.

### Propagation of F. graminearum

The *F*. *graminearum* strain used in the plant trials was Z3639 [44], generously provided by Dr. Susan McCormick (USDA). Mycelium were grown on potato dextrose agar (PDA) plates with 50 μg/mL streptomycin-sulfate at room temperature with natural lighting. For spore production, cultures were grown in carboxymethylcellulose (CMC) media with 50 μg/mL streptomycin-sulfate (1.0 g NH4NO3, 1.0 g KH2PO4, 0.5 g MgSO4-7H2O, 1.0 g yeast extract, 15.0 g CMC up to 1 L water). Cultures were incubated at 27°C,180 rpm for 4–7 days. The cultures were shaken aggressively to release macroconidia from the mycelia, and filtered through four layers of cheese cloth. Spores were collected by centrifugation, washed three times with sterile water and adjusted to 5*10^4^ macroconida mL^-1^. Two central spikelets were point inoculated with 10 μL of spore suspension at anthesis [45]. Plants were transferred to a high humidity (> 95%) chamber for three days to initiate Fusarium infection.

### Barley Stripe Mosaic Virus-based Virus Induced Gene Silencing

Primers for the amplification of a 217 base pair portion of the Ta_PYL2AS_FL cv. ‘Chinese Spring’ gene were designed using online Primer3 software [46, 47]. Ligation Independent Cloning (LIC) sites were manually added to the primer sequences afterwards yielding Ta_2AS_FL-F: 5’-AAGGAAGTTTAAGCTGGAGATCCTGGACGAC-3’ and Ta_PYL2AS_FL-R: 5’-AACCACCACCACCGTGTTGCACTTGACGATGGTGT-3’. All primers were synthesized at the DNA synthesis lab at the National Research Council in Saskatoon, SK, Canada. Following PCR amplification from cDNA isolated from *T*. *aestivum* cv. ‘Fielder’, the amplicon was purified using the QIAquick PCR purification kit (Qiagen) and cloned using the LIC method previously described [48], with some modifications. The barley stripe mosaic virus (BSMV) γLIC plasmid was modified to carry a lethal ccdB cassette (BSMVγLIC-ccdB) The construct was digested with ApaI (New England Biolabs) and extracted with phenol:chloroform:isoamyl alcohol (25:24:1) [49]. The T4 DNA polymerase treatment of BSMVγLIC-ccdB was carried out by adding together 4 μL digested plasmid, 2 μL NEB buffer 2, 1 μL dTTP (100 mM), 1 μL DTT (100 mM), 0.2 μL BSA (1000 x), 0.4 μL T4 DNA polymerase and bringing the final volume to 20 μL with sterile water. The PCR product was treated in a similar manner with 1 μL dATP (100 mM) added instead of dTTP. Both reactions were incubating at room temperature for 30 min followed by a 20 min incubation at 75°C to inactivate the polymerase. The LIC cloning reaction was performed by mixing 1 μL vector DNA, 1 μL insert DNA, and 3 μL water together, incubating the tube at 66°C for 2 min and then allowing the reaction to cool at room temperature for 20 min. After bacterial transformation colonies were selected on 50 μg/mL kanamycin LB media and confirmed by sequencing. The plasmid (BSMVγLIC-PYL4AS_A) was transformed into *Agrobacterium* using electroporation and grown on LB plates containing 50 μg/mL kanamycin and 10 μg/mL rifamycin.

The virus induced gene silencing (VIGS) virus was passed through a *Nicotiana benthamiana* intermediate for amplification. BSMVγLIC-PYL4AS_A, BSMVγLIC-GFP, BSMVβ, BSMVα, and P19 *Agrobacterium* cell lines were used to inoculate separate 5 mL volumes of LB media and incubated overnight at 28°C. Cultures were centrifuged at 4500 x g for 10 minutes and pellets were resuspended to an OD600 of 0.700 in infiltration buffer (10 mM MgCl2, 10 mM 2-(N-morpholino)-ethanesulfonic acid (MES) (pH 5.2), and 0.1 mM acetosyringone). Equal volumes of α, β, γPYL4AS_A/γGFP, and P19 mixed and incubated at room temperature for 4 hours. A 1 mL needleless syringe was used to push the mixture into the abaxial surface of the leaves of 4 week old *N*. *benthamiana* plants. The *N*. *benthamiana* plants were left to accumulate the virus in the leaves for 1 week. Infiltrated leaves from VIGS TaPYL4AS_A and GFP infected *N*. *benthamiana* plants were ground in phosphate buffer (20 mM sodium phosphate monobasic hydrate (pH 7.2)), silica sand and silicon carbide. Wheat plants were inoculated by pinching and rubbing the *N*. *benthamiana* leaf extracts on the flag leaf of each tiller 3–5 days before the emergence of the head.

### Wheat Disease Phenotyping

The progression of the FHB disease in knock-down and control plants was measured at 3, 5, 7, 9, and 13 days post infection. The number of infected spikelets per head as well as the number of infected rachis nodes per head wasquantified at each time point. Measurements were made from 3–6 tillers per plant. Any tillers that developed late or beyond the six maximum wereremoved. Plants infiltrated with either Ta_PYL4AS_A or GFP VIGS vector constructs were divided evenly into treatment groups infected with Fusarium and uninfected control groups. The number of infected spikelets per head was determined using all available heads in the respective treatment or control group (n = 9). Spikelet and rachis data were analyzed using 2-Way ANOVA to determine the effect of the VIGS construct and Fusarium inoculation (p < 0.05). When a significant interaction between the VIGS construct and Fusarium infection was observed, a Student’s t-test was used to determine differences between VIGS constructs at each day of infection (Data analysis add-in, Microsft Excel 2013). Whole heads were collected for DON analysis. Spikelets immediately underneath the initial Fusarium inoculation site were collected for qPCR and RNA SEQ analysis. All samples were snap frozen in liquid nitrogen and stored at -80°C until further analysis.

### qPCR

Plant samples were taken at 7 days post Fusarium infection (FG; 14 days after inoculation with VIGS constructs). *Ta_PYL4AS_A* levels were measured in Ta_PYL4AS_A, Ta_PYL4AS_A-FG, GFP, and GFP-FG plants. Primers were designed using the online Primer3 software [46, 47]. The primer set used for the target was Ta_PYL4AS_A-UTR4_F: CCGTGTCGTGACTCCAGTC, and Ta_PYL4AS_A-UTR4_R: CGCCGAAGAAACACACATCC. This primer set was outside of the VIGS construct region and targeted the 3’ un-translated region of the gene to decrease the level of non-specific amplification. The endogenous control gene used was ACTII (Actin) with the primer set EW412: CAAATCATGTTTGAGACCTTCAATG and EW413: ACCAGAATCCAACACGATACCTG. All primers were synthesized at the DNA synthesis lab at the National Research Council of Canada in Saskatoon. To extract total RNA, the RNeasy Plant Mini Kit was used with the RNase-Free DNase kit following the manufacturer’s instruction (Qiagen). RNA was reverse transcribed into cDNA using the QuantiTect Reverse Transcription Kit (Qiagen) following manufacturer’s instructions. The qPCR analyses from three technical replicates were carried out using a StepOnePlus Real Time PCR instrument and accompanying software (Applied Biosystems). qPCR reactions were prepared using the Fast SYBR Green kit (Applied Biosystems) following manufacturer’s instructions. The PCR conditions were; 95.0°C for 5 minutes followed by 40 cycles at 95.0°C for 20 s and 60°C for 30 s. A melting curve was included for analysis and negative controls using water and RNA were include in each experiment. The effect of VIGS inoculation and FG infection was determined from the technical replicates, using 2-Way ANOVA. When a significant interaction between the VIGS construct and Fusarium infection was observed, a Student’s t-test was used to determine differences between infected and uninfected plants for each VIGS construct.

### DON quantification

Ta_PYL4AS_A and Ta_PYL4AS_A-FG, whole head samples (n = 3) were harvested at five and thirteen days past Fusarium infection and ground in liquid nitrogen with an autoclaved mortar and pestle. DON was extracted with 4 mL/g 84% acetonitrile-water extraction solvent by incubating at 1000 rpm for 2 hours at room temperature. Samples were filtered through Whatman No 2 filters and stored in capped brown vials at 4°C until analysis. An aliquot of the extract was diluted 10 fold with 84% acetonitrile-water and DON levels were measured using a Waters LC/MS as previously described [50]. Differences between samples at day 5 and day 13 were determined separately for each day using a Student’s t-test.

## Results

### Identification of putative *T*. *aestivum* and *B*. *distachyon* ABA receptor gene families

Toward investigating ligand specificities of monocot ABA receptors, we searched for *B*. *distachyon* (as a fully sequenced model system) and *T*. *aestivum* (as a draft sequenced crop system) ABA receptor homologs from existing genomic databases. On the side of *B*. *distachyon* a BLAST search of the *B*. *distachyon* genomic sequences (www.plantgdb.org/BdGDB/) using the *Arabidopsis* AtPYL5 (accession NP_196163) cDNA sequence as the query sequence, yielded 9 unique sequences encoding putative full length ABA receptors, all of which are annotated as ABA receptor-like proteins (Table 1 and Table A in S1 File). In the case of *T*. *aestivum*, initially the *Arabidopsis* AtPYL5 cDNA sequence was also used as a blast query against all available wheat sequences in the NCBI database. At the time, two putative wheat PYL cDNAs were identified from *T*. *aestivum* cv. ‘Chinese Spring’ (bread wheat; accessions AK335719 and CDM82373). The coding region of the AK335719 was predicted to yield a protein of 214 amino acids, with 59% amino acid sequence identity and over 85% sequence coverage, to the *A*. *thaliana* AtPYL5 receptor, while CDM82373 is 201 amino acids long, and 53% identical with 95% coverge of AtPYL5 (Table 1, Table A in S1 File and Figure A in S2 File).

The cDNA for AK335719 was subsequently used as a template to search the wheat (*T*. *aestivum* cv ‘Chinese Spring’), International Wheat Genomics Sequencing Consortium (IWGSC) survey sequence and wheat reference sequence chromosome 3B databases by blastn and tblastn (https://urgi.versailles.inra.fr/blast/; [51]). At the time of writing, portions of twenty unique putative wheat ABA receptors were identified in the *T*. *aestivum* genome (Table 1 and Table A in S1 File). Additional searches using *B*. *distachyon* and *A*. *thaliana* receptor sequences here did not yield any additional hits. Until the full genomic sequence of wheat is available, and the full complement of full-length ABA receptors can be identified, no ‘common’ names are being assigned to these putative wheat ABA receptor hits. Instead, wheat sequences are identified throughout the remainder of this report by genomic localization according to the current database (e.g. Ta_PYL3AS refers to that putative receptor identified from the sequence data available for the short arm of chromosome 3 of the *T*. *aestivum* A genome). Predicted amino acid sequences for both the *B*. *distachyon* and *T*. *aestivum* were aligned with *A*. *thaliana* AtPYL5 using Clustal Omega (Table A in S1 File and Figure A in S2 File). As expected the annotated *B*. *distachyon* sequences were all found to be full length and their amino acid identities to AtPYL5 ranged from approximately 40–65%. On the side of wheat, the sequences were found to be less complete, with many hits yielding only 40–90 amino acid long segments. Amino acid identities with respect to the AtPYL5 sequence ranged from 47–68%; although the partial nature of many of these hits must be taken into consideration when interpreting these values. Notably, sequences identified on chromosomes Ta_1AS, Ta_1BS and Ta_1DS are almost, but not quite identical and may likely represent the homeoelogous alleles of the same gene in A, B, and D chromosomes. Similarly, the set of chromosome 4 genes, Ta_PYL4AS_A, Ta_PYL4BL and Ta_PYL4DL as well as the set of chromosome 7 gene Ta_PYL7AL, Ta_PYL7BL and Ta_PYL7DL, are respectively also close to identical and may also represent homeoelogous alleles, although this awaits validation. The NCBI cDNA AK335719 sequence shows 100% amino acid (and DNA) identity with the receptor fragment (79 amino acids) identified on wheat chromosome 2DS (Ta_2DS; Table 1 and Figure A in S2 File) and is referred to as Ta_PYL2DS_FL for the remainder of this report. Interestingly, Ta_PYL2BS_A shows only a single amino acid difference (within its limited range) to Ta_PYL2DS_FL (as opposed to Ta_PYL2BS_B that shows 16 amino acid variations within this region), and thus may represent a homologous allele here, although this remains to be confirmed when full sequences are available. Indeed while Ta_CDM82373 is identical at the cDNA and amino acid levels to Ta_PYL2BS_A, it is also identical to Ta_PYL3B_B, so that it is not possible to match it definitely at this time..

**Fig 1:**
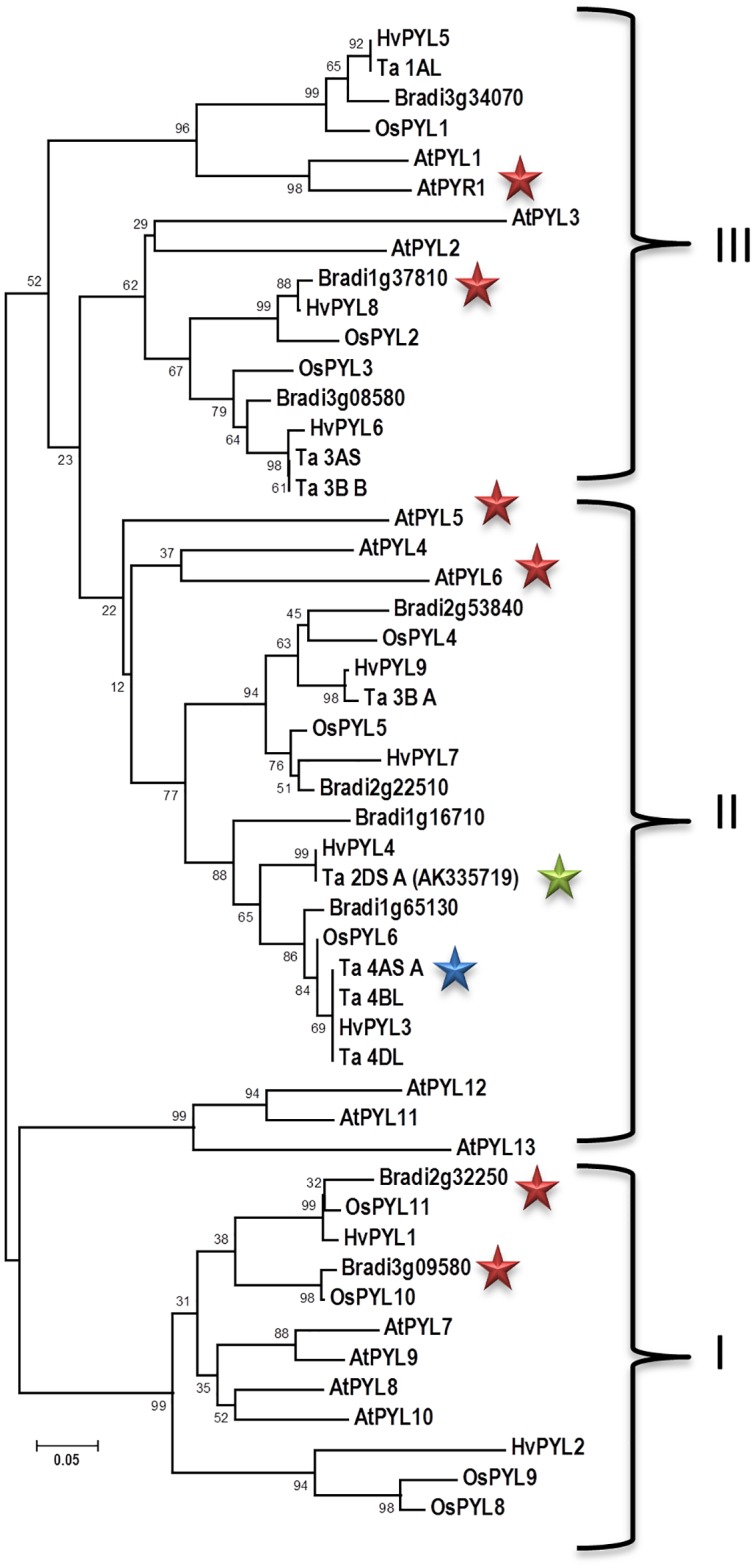
Evolutionary relationships of wheat, rice, *B*. *distachion* and *A*. *thaliana* ABA receptors. The evolutionary history was inferred using the Neighbor-Joining method [53]. The optimal tree with the sum of branch length = 4.66070389 is shown. The percentage of replicate trees in which the associated taxa clustered together in the bootstrap test (500 replicates) are shown next to the branches [54]. The tree is drawn to scale, with branch lengths in the same units as those of the evolutionary distances used to infer the phylogenetic tree. The evolutionary distances were computed using the Poisson correction method [55] and are in the units of the number of amino acid substitutions per site. All positions containing gaps and missing data were eliminated yielding a total of 79 positions in the final dataset. Evolutionary analyses were conducted in MEGA6 [41]. Gene locator ID’s are included in Table 1, and for barley and rice [21, 22].

To further investigate these relationships, a phylogenetic analysis was carried out based on a sequence alignment of the nine putative *B*. *distachyon* sequences and the nine *T*. *aestivum* sequences that had greater than 50% coverage of the AtPYL5 sequence. The analysis was limited to these sequences as inclusion of shorter fragments diminished the reliability of the alignment and phylogenetic outputs significantly. Together these were evaluated against the previously published rice, barley and *A*. *thaliana* ABA receptor gene families [19–22]. As expected the *A*. *thaliana* ABA receptors fell into three previously defined subfamilies [2, 52], with the rice, barley and putative *B*. *distachyon* receptors also falling in all three of these subfamilies as well (Fig 1). In contrast, none of the putative *T*. *aestivum* ABA receptors included in the analysis fell into subfamily I, but did have representatives in both of subfamilies II and III (Fig 1). Consideration of the clustering of both rice and *B*. *distachyon* genes in subfamily I, it should be expected that some of the currently identified wheat fragments may likely fall into this subfamily when their full sequence is available and analyzed. The close relationship of the chromosome 4 genes (Ta_PYL4AS_A, Ta_PYL_4BL and Ta_PYL_4DL) is further emphasized by their clustering in subfamily II along with Bradi1g65130 and OsPYL6. As well, the likelihood that Ta_PYL3AS and Ta_PYL_3B_B represent homologous alleles in the A and B genomes on chromosome 3 is highlighted by their clustering, along with Bradi3g08580 and OsPYL3. Unfortunately the Ta_PYL2BS_A gene fragment was too short to be included in the phylogentic analysis relative to Ta_PYL2DS_FL. Finally, none of the monocot receptors clustered with AtPYL11, AtPYL12 or AtPYL13 genes, highlighting a division within subfamily II that was not apparent in the initial phylogenetic analysis on *Arabidopsis* receptors alone.

### Ta_PYL2DS_FL (AK337519) is confirmed to have ABA receptor activity *in vitro*

Ta_PYL2DS_FL was selected for further investigation based on its existing NCBI listing as an expressed mRNA. A cDNA encoding codon-optimized putative ABA receptor Ta_PYL2DS_FL as well as a non-codon optimized wheat PP2C phosphatase TaABI1 (accession AB238930.1) [42], were synthesized and cloned into expression vectors for recombinant expression in *Escherchia coli*. The resulting N-terminally His-tagged proteins were enriched by Ni-NTA affinity purification and the ability of the recombinantly produced Ta_PYL2DS_FL to inhibit TaABI1 in an ABA-dependent manner *in vitro* was assessed. TaABI1 was shown to have strong phosphatase activity in the presence of Ta_PYL2DS_FL, which was reduced by almost 90% upon the addition of ABA (Figure B in S2 File). This confirms that Ta_PYL2DS_FL is an ABA receptor capable of inhibiting the TaABI1 proteins in an ABA-dependent manner *in vitro*.

### *T*. *aestivum* Ta_2DS_FL shows unique ligand selectivity compared to *A*. *thaliana* ABA receptor homologs

Subsequently the activities of the *T*. *aestivum* Ta_PYL2DS_FL receptor and TaABI1 PP2C were compared to recombinantly produced and enriched *A*. *thaliana* AtPYL5 (accession NP_196163, a well-studied homolog in subfamily II) and PP2C AtABI1 (accession numbers: CAA54383), toward differentiating the activities of these receptors.

**Fig 2:**
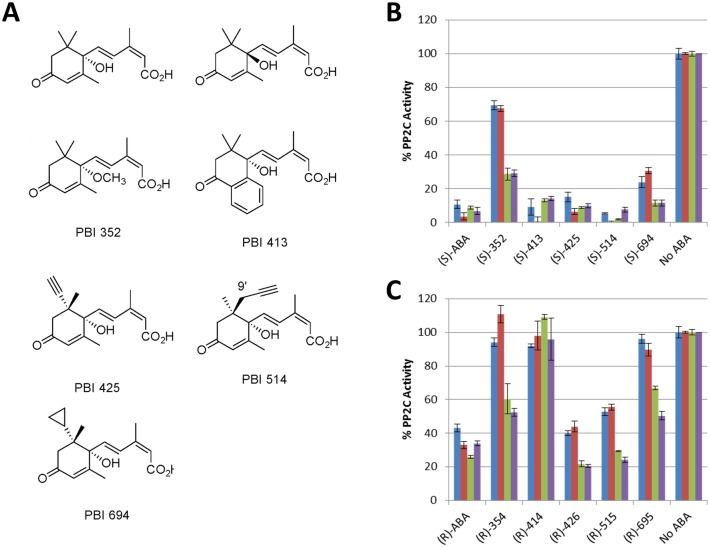
The ABA Analog Activity Profiles of Ta_PYL2DS_FL are different than AtPYL5. AtABI1 and TaABI1 (AB238930.1) activity is regulated by wheat Ta_PYL2DS_FL and Arabidopsis AtPYL5 ABA receptors against various enantiomeric ABA analog pairs. A) The structures for (+)- and (-)- ABA, as well as (+)-analogs are shown. The corresponding (-)-analogs are not shown. B) Activities in the presence of *(S) or (+)–*enantiomers; C) Activities in the presence of *(R)***- or (-)** enantiomers. The protein phosphatase activity was analyzed at a constant molar ratio of receptor to PP2C of 10:1 *in vitro* at a constant analog concentration of 0.1 μM (n = 3). Blue and Red bars show activity for Ta_PYL2DS_FL against TaABI1 and AtABI1 respectively. Green and Purple bars show activity for AtPYL5 against AtABI1 and TaABI1 respectively.

With recombinant proteins in hand, all permutations of receptor-PP2C combinations were tested using not only the native receptor ligand *(S)- or* (+)-ABA, but also a panel of ABA-like analogs including *(R)- or (-)-*ABA and five other enantiomeric pairs of ABA-like analogs (Fig 2A). These were recently used in a study probing the structure-activity relationships of the thirteen active *A*. *thaliana* ABA receptors and four *A*. *thaliana* PP2Cs *in vitro*, relative to analog functionality *in planta* during development and stress response [38]. These five (+) analogs along with their (-) enantiomers have single site changes to the ABA molecule. Analogs PBI352 and PBI354 are (+) and (-) enantiomers respectively that have the proton of the hydroxyl group replaced with a methyl group. Analogs PBI413 and PBI414 are (+) and (-) enantiomers that have the 7’ methyl replaced with an aromatic ring fused to the 3’ and 2’ carbons of the ABA ring. Analogs PBI425 and PBI426 are (+) and (-) enantiomers that have the 8’ methyl group replaced with an acetylene group. Analogs PBI514 and PBI515 are (+) and (-) enantiomers that have the 9’ methyl group replaced with a propargyl group, and finally analogs 694 and 695 are (+) and (-) enantiomers that have the 8’ methyl group replaced with a cyclopropyl group.

On this basis, Ta_PYL2DS_FL and AtPYL5 were directly compared for their ability to inhibit both TaABI1 and AtABI1, in the presence or absence of ABA and versus the panel of ABA-like analogs. Generally the four receptor-PP2C combinations yielded similar profiles of activities. Similar to a previous report for *A*. *thaliana* receptors [38], *(S)-*ABA and related (+)-ABA-like analogs were potent *in vitro* acting on all receptor-PP2C combinations tested here, yielding approximately 80–90% reductions in PP2C activity (Fig 2B). Potencies of the (-)-analogs were generally weaker at the same given analog concentrations compared to the (+)-analogs, although some PP2C inhibition was still detected in almost all cases (Fig 2C). However some exceptions to these general trends are noted. One of the most obvious is the differential effect the methyl ether substituted PBI352/PBI354 enantiomeric pair, has on the wheat receptor compared to the *A*. *thaliana* receptor. In particular when applied to AtPYL5, both of these analogs yield 50–70% decreases in PP2C activity, regardless of the PP2C in the reaction. In contrast, application of these analogs to the wheat Ta_PYL2DS_FL receptor yielded only a 30% decrease in PP2C activity for PBI352, while PBI354 had no detectable effect (0% decrease in PP2C activity) on either PP2C. Similar effects, albeit it not as pronounced, are observed for the PBI694/PBI695 enantiomeric pair of analogs, as well as two of the more potent (-)-analogs including PBI426 and PBI515.

Toward further investigating the significance of the differential responses to select analogs including PBI352, two additional *A*. *thaliana* receptors AtPYL6 and AtPYR1 (accession numbers: Q8S8E3; O49686; red stars Fig 1) and three *B*. *distachyon* receptors Bradi’s 2g32250, 3g09580 and 1g37810 (accession numbers: XP_003568737, XP_003572108 and XP_003563747 respectively; red stars Fig 1) were recombinantly produced and enriched as described above, yielding an array of receptors for analysis spanning the phylogenetic tree (Fig 1, colored stars; two receptors per phylogenetic subfamily). *A*. *thaliana* PP2C’s AtABI2 and AtHAB1 (accession numbers: CAA72538 and Q9CAJ0 respectively) as well as Bradi2g41950, which showed 43% identity to AtABI1, were also prepared as described for the wheat PP2C, and used in *in vitro* assays.

The data arising from evaluation of the three *B*. *distachyon* receptors against PP2C Bradi2g41950 and the three *A*. *thaliana* receptors against the Ta_ABI1 PP2C were plotted (Figure C in S2 File). Again potency was generally higher for the (+)-analogs compared to the (-)-analogs, and it might be argued that responsiveness of receptors across phylogenetic subfamilies is generally I > II > III. None-the -less it is interesting to note that the analog response profile for the subfamily III receptor Bd1g37810 is similar to that of the subfamily II, Ta_PYL2DS_FL. At the same time, AtPYR1 appears to be uniquely insensitive across the analog profile. Likely this latter effect is linked to a previously characterized dimerization mechanism exclusively associated with select members of the subfamily III receptors [56]. A few other exceptions to the general trends are also noted. PBI541 is lacking in potency against receptor Bd3g09580, and similarly PBI414 is lacking in potency against AtPYL5 compared to the other receptors (except AtPYR1). In the case of PBI352 the subfamily trend of I > II > III is especially notable with inhibition of PP2Cs ranging from 90% (*B*. *distachyon* subfamily 1 receptors), to no effect at all (AtPYR1), and highlights that the differential effect observed between AtPYL5 and Ta_PYL2DS_FL (Fig 2) is not especially unique. Indeed PBI352 seems to elicit especially diverse degrees of responses against different receptors. This emphasizes a uniquely selective analog that was also previously shown to elicit selective physiological effects *in planta*, stimulating stomatal closure, but having no effect on root growth or inhibition of germination [38]. Thus here we further validate that analog PBI352 is unique and that it’s mechanism of receptor selectivity and functional effects should be further investigated. Finally, to account for the possibility that some of the differences observed here might be arising due to the use of different PP2Cs, a final experiment was conducted applying Ta_PYL2DS_FL against three different *A*. *thaliana* PP2Cs and the one wheat PP2C (Figure D in S2 File). In contrast to the effect of varying receptor, varying PP2C had little impact, highlighting that generally the selectivity of the analogs is linked to its interaction with the receptor and not the PP2C, but also highlighting little effect in the pairing of a receptor with different PP2Cs. One exception is that the AtHAB1 was selectively more sensitive to the receptor, emphasizing that one can not make assumptions, although the effect is relatively weak. That the *B*. *distachyon* data arose from a high throughput screening study with n = 1 must be kept in mind when interpreting this data.

### Amino acid reside 180 is involved in mediating the weaker effect of analog 352 against Ta_PYL2DS_FL compared to AtPYL5

**Fig 3:**
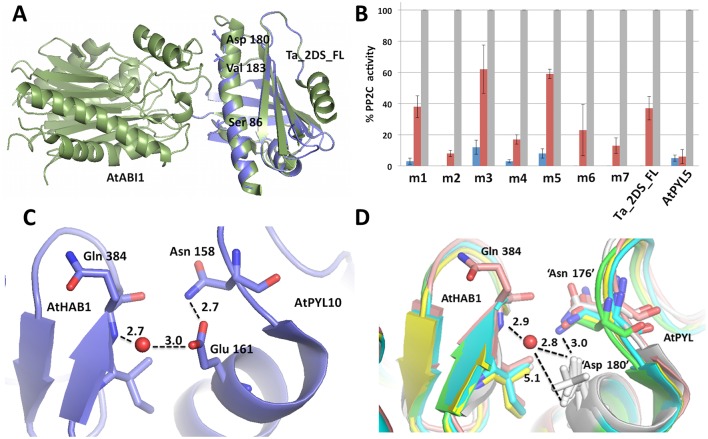
Amino acid reside 180 is involved in mediating the weaker effect of analog 352 against Ta_PYL2DS_FL compared to AtPYL5. A) Homology model of Ta_PYL2DS_FL (blue) was generated using the translated Genebank AK335719 sequence and a template structure of AtPYL1 (PDB ID 3NMN chain A) co-crystallized in complex with AtPP2C using the SWISS-MODEL Workspace [59]. Favorability of this model is supported based on 56% sequence identify, alignment of all substrate-binding residues to the template sequence, and a Global Model Quality Estimation scores of 0.72. This method was also applied to AtPYL5 (green aligned protein) using the NCBI NP_196163.1 sequence and yielding a GMQE score of 0.68 over the 52% identical sequences. The co-crystallized PP2C structure, At ABI1 (green) is included from the original co-crystallized template structure. B) The activity of Ta_PYL2DS_FL variants against ABA and PBI352. The activity of TaABI1 is differentially regulated by variants of Ta_PYL2DS_FL compared to WT receptor and AtPYL5, in the presence of (+)-ABA (blue bars) and analog PBI352 (red bars). The total TaABI1 activity arising in the absence of receptor stimulation was set to 100% (grey bars). The variants tested included: m1 (S86N), m2 (D180E), m3 (V183S), m4 (D180E + V183S), m5 (S86N + V183S), m6 (S86N + D180E), m7 (S86N + D180E + V183S). The protein phosphatase activity was analyzed at a constant molar ratio of receptor to TaABI1 of 10:1 in vitro at a constant analog concentration of 0.1 μM. Error bars show the standard deviation (n = 3). C) Crystal structure of AtPYL10 in complex with AtHAB1 (PDBID 3 3RTO), showing the dual hydrogen-bonding interaction of E161 (equivalent of Glu180 in AtPYL5) with AtHAB1 Q384 and AtPYL10 N158. D) Crystal structures of PYR1+HAB1 (4WVO; light teal), PYL1+AB11 (3NMN; green), PYL2+ABI2 (3UJL; yellow), PYL3+HAB1 (4DS8; pink), PYL2+HAB1 (4LG5; white) overlaid showing the interaction of the conserved ‘D180’ residues (equivalent to D180 in Ta_PYL2DS_FL) in these structures. The shorter D side chain (compared to E in AtPYL10) limits the residue to making only one hydrogen-bond interaction at a time, likely yielding a weaker overall receptor-PP2C interaction. All overlays and structure images were produced using PyMOL Molecular Graphics System, Version 1.8 Schrödinger, LLC.

Toward further investigating the structure-activity relationships that define the selective interactions with PBI352 in the Ta_PYL2DS_FL-AtPYL5 comparison, a sequence comparison of the two receptors was completed taking into special consideration residues previously predicted to mediate all of the receptor-ligand-PP2C interactions [57]. Of the 33 residues implicated in forming these regions, only three amino acids were found to vary between Ta_PYL2DS_FL and AtPYL5 (Figure E in S2 File). These include S86/88N, D180E, and V183S (notated as wheat/*A*. *thaliana*; numbering is the same at sites 180 and 183 for both receptors, site 86 in wheat is the equivalent of site 88 in *A*. *thaliana* as indicated). Interestingly, none of these three sites are predicted to contribute to making interactions with ABA, but rather all lie in regions including the loop between β-strand 1 and helix 2 (S86N) and the final helix 5 (D180E and V183S) contributing to forming the PP2C-receptor interface (Fig 3A) [57–58]. This might suggest that the variations in responses to PBI352 are not arising strictly due to changes associated with binding of the ligand to the binding site. Rather the PBI352 analog, once bound, may differentially modulate the ligand-stimulated conformational changes associated with creating the receptor-PP2C interface, potentially leading to decreased PP2C binding affinity, due to the amino acid variations. That both receptors have similar activity in the presence of *(S)-*ABA suggests that it is a combination of the PBI352 analog and the amino acid variations leading to the effect.

To assess the role of these three variable amino acid sites in modulating receptor activity with respect to stimulation by the PBI352 analog, site directed mutagenesis was carried out to incorporate the AtPYL5 amino acids into the Ta_PYL2DS_FL receptor including S86N, D180E and V183S in all possible permutations and combinations (including single, double and triple mutations). The ability of the resulting variants to inhibit TaABI1 activity *in vitro* was assessed (Fig 3B). Initially looking at the 3 single site mutations, S86N (m1), D180E (m2), V183S (m3), an array of effects is observed. Most notably D180E led to a dramatic increase in receptor mediated inhibition of the phosphatase in the presence of PBI352, such that the activity of this variant matches that of AtPYL5. In contrast S86N had no effect, while V183S on its own led to a decrease in PBI352-receptor mediated inhibition, although this last effect suffers from high variability. Combining the variations into double and triple variants show that S86N + V183S (m5) together still do not improve the response to PBI352 and like V183S alone, may actually make it worse. However combining D180E with any of the other mutations, D180E + V183S (m4), S86N + D180E (m6), S86N + D180E + V183S (m7), again yielded activity almost comparable to AtPYL5, although notably the D180E+V183S mutation (m4) was not as effective as the others. Overall this data highlights a critical role for the D180E site in mediating the differential receptor responses to the PBI352 analog. Consistent with this, Bd1g37810 which had an analog response profile similar to that of Ta_PYL2DS_FL, contains an Asp (D) at its site equivalent to the D180 site (Figures A and C in S2 File).

A comparative investigation of the structures of the six available *A*. *thaliana* PYL/PP2C complexes (including PYR1+HAB1 (4WVO), PYL1+AB11 (3NMN), PYL2+ABI2 (3UJL), PYL3+HAB1 (4DS8), PYL2+HAB1 (4LG5) and PYL10+HAB1 (3RTO)), shows that all of the interfaces adopt the same fold and make many of the same interactions. The amino acid site of interest (D180 in TA_PYL2DS_FL; E180 in AtPYL5) is located near the N-terminus of the C-terminal receptor helix, known to make critical interactions with the PP2C (Fig 3A and 3C; [58]). In one of the six structures (PYL10+HAB1), an E is present at the equivalent position (E161 in AtPYL10; equivalent with E180 in AtPYL5). The E at this position easily makes two hydrogen-bonds, the first to a conserved N158 (i-4) that is situated in the loop just before the start of the terminal helix in the receptor, as well as a second water mediated H-bond with the PP2C at amino acid Q384 (Fig 3D). At the same time the Ta_PYL2DS_FL ‘D180’ variation is conserved in five of the available structures (including receptors PYR1, PYL1, PYL2 and PYL3). While ‘D180’ can still form a H-bond with either the conserved N at position 158 in the receptor, or the water mediated H-bond to the PP2C, the shorter side chain precludes making both at the same time, such that the overall interaction with the PP2C will be weaker. Thus E180 in AtPYL5 is likely contributing a stabilizing water mediated hydrogen bond interaction with the PP2C, that is significantly weaker or even lacking completely in the D180 variants, as in Ta_PYL2DS_FL.

### Knockdown of Ta_PYL4AS_A and other close homologs in wheat leads to increased resistance to Fusarium head blight

While classically associated with developmental effects and responses to abiotic stress, the role of ABA with respect to disease responses is increasingly being reported in the literature (Cao et al, 2011). Most recently two papers have shown that priming of wheat heads with ABA increases susceptibility to FHB [33, 34]. To further evaluate the mechanism of ABA action on wheat disease, VIGS was used to knockdown putative ABA receptor(s). Primers were designed to amplify a 217 bp segment of the Ta_PYL2DS_FL receptor for cloning into the BSMV vector system [48] based on the Ta_PYL2DS_FL sequence from ‘Chinese Spring’ (Figure F in S2 File). However the fragment was amplified from the *T*. *aestivum* ‘Fielder’ as this variety was used for the knockdown experiments. Interestingly, it was a homolog of the Ta_PYL4AS_A gene that was amplified from this background using the ‘Chinese Spring’ Ta_PYL2DS_FL primers (Figure F in S2 File).

**Fig 4:**
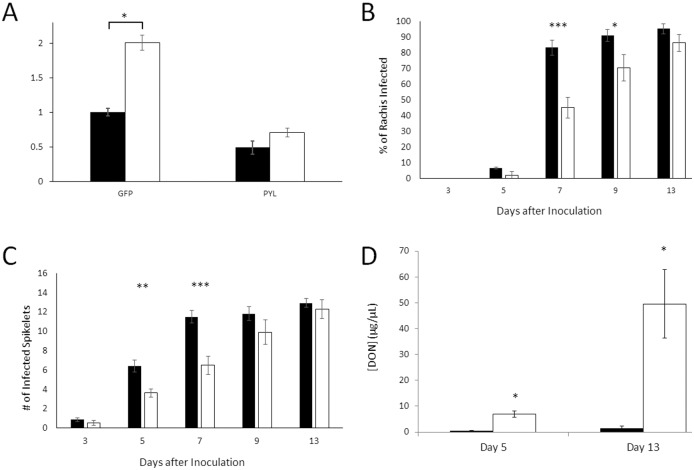
Effect of the VIGS knockdown of Wheat Ta_PYL4AS_A on *F*. *graminearum* infection of *T*. *aestivum* cv. ‘Fielder’. A) Relative expression of Ta_PYL4AS_A in various VIGS treated wheat plants. Quantification of the gene expression of Ta_PYL4AS_A was performed 12 days post BSMV:Ta_PYL4AS_A application (+/- *F*. *graminearum* (Fg) application 7 days after the BMSV rub), compared to controls treated with BMSV:GFP, with Ta_actin used as an endogenous control. Uninfected plants, black bars; Fg infected plants, white bars. The expression level of the BMSV:GFP control was set to a value of 1 (n = 3). BSMV treated plants were treated with *F*. *graminearum* spores 7 days after the BMSV rub and assessed for *F*. *graminearum* infection in the B) spikelets and C) rachis at days 3, 5, 7 9 and 13 post—*F*. *graminearum* infection in BSMV:GFP (black bars) and BSMV:Ta_PYL4AS_A (white bars). Data were tested using 2-way ANOVA, and for each day separately using a t-test. (n = 9). D) Accumulation of DON in BSMV:Ta_PYL4AS_A (black bars) and BSMV:GFP (white bars) treated wheat heads. Samples were analyzed by LC/MS at 5 and 13 days post *F*. *graminearum* infection (n = 3). For all panels, error bars indicate standard error of the mean. Significant differences between samples are indicated with asterisks, (* p < 0.05, ** p < 0.01, *** p < 0.001).

The 217 bp fragment of Ta_PYL4AS_A was cloned into the VIGS BSMV vector system, and applied to wheat as described previously [45]. The effect of infecting plants with the BMSV:Ta_PYL4AS_A vector on Ta_PYL4AS_A gene expression was assessed by qPCR, showing a significant reduction in Ta_PYL4AS_A expression in the presence of BSMV:Ta_PYL4AS_A (approximately 2-fold lower) than those infected with BMSV:GFP (containing a fragment of a green fluorescent protein;Figure F in S2 File) at 12 days post BSMV application (Fig 4A). There was a significant effect of both the VIGS construct and *F*. *graminearum* infection, as well as an interaction between these factors. Interestingly, when BSMV-treated plants were subsequently inoculated with *F*. *graminearum* spores (at day 7 after BSMV application), Ta_PYL4AS_A gene expression was increased > 2 fold in the BSMV:GFP-control plants. Uninfected BSMV:Ta_PYL4AS_A expressing plants had approximately 50% less transcript than the controls, and showed only a 40% increase in response to *F*. *graminearum*, that was not significantly different from the un-inoculated control (Fig 4A). This indicates successful knockdown of the target gene (and close homologs thereof) by the BSMV:Ta_PYL4AS_A construct.

With VIGS knockdown of some compliment of ABA receptors homologous to Ta_4AS_A confirmed by qPCR, the effect of the knockdown on FHB infection and accumulation of the mycotoxin DON were assessed. There was a significant effect of both VIGS construct and infection, as well as a VIGS x infection interaction for both number of spikelets infected as well as spread throughout the rachis. In particular the BSMV:Ta_PYL4AS_A expressing plants showed reduced numbers of infected spikelets on days 5 and 7, and a decreased infection spread through the rachis on days 7 and 9, compared to the BSMV:GFP expressing control plants (Fig 4B and 4C). The effect of ABA-receptor knockdown on the accumulation of pathogen-derived mycotoxin DON was assessed at 5 days and 13 days post F. *graminearum* infection. In both cases expression of the BMSV:Ta_PYL4AS_A construct significantly reduced the amount of DON accumulating, by 5-fold at 5 days post *F*. *graminearum* inoculation and up to 25-fold by 13-days post *F*. *graminearum* (Fig 4D). It is important to note that the apparent rate of FHB infection documented here is somewhat faster than normal. This effect in BSMV treated plants is well documented and related to an interaction between BSMV and *F*. *graminearum* infection causing an apparent hyper susceptibility to the fungus in wheat [45].

## Discussion

Together this work has yielded the identities of a number of *T*. *aestivum* and *B*. *distachyon* ABA receptors, highlighted that residue D180 in Ta_PYL2DS_FL is a critical determinant in translating selective ABA analog stimulation downstream via the PP2C interaction, and demonstrated a role for the wheat receptor Ta_PYL4AS_A (and close homologs) in mediating susceptibility to *F*. *graminearum* infection.

Deciphering unique physiological roles for individual ABA receptors, and broad differences between phylogenetic clades is ongoing. Early studies showed that deletion of as many as four receptors (from across the phylogentic clades) was necessary to elicit a physiological response [60], emphasizing genetic redundancy. At the same time, while a number of receptor-over expression studies did link select receptors to specific biological functions, as time passes and more reports become available, it is clear that multiple ABA receptors are able to carry out the same functions [61–65]. In this vein, one recent effort focusing on the unique Clade III receptors that exist naturally as dimers, successfully demonstrated that these are all key mediators of water use efficiency by targeting them with a selective agonist [66]. More recently, a larger panel of ABA-analogs was used to comparatively screen the full-breadth of *A*. *thaliana* ABA receptors *in vitro*, highlighting unique interactions that differentiate the receptors [38]. *In planta* one uniquely selective analog (PBI352) that was most effective at stimulating Clade II receptors including AtPYL5 was found to mediate stomatal closure, but not inhibition of root growth or seed germination, linking this receptor clade to stomatal function.

Herein, with respect to the VIGS knockdown of Ta_PYL4AS_A, it must be noted that it is unlikely that only Ta_PYL4AS_A was knocked down in the VIGS experiment, with similar genes also silenced (Table 1). Indeed as one might expect for a hexaploid system, cDNA alignments of the VIGS insert with the other two chromosome 4 hits (Ta_4BL and Ta_4DL) shows 95 and 96% cDNA identity respectively with Ta_PYL4AS_A in the VIGS insert region (Figure G in S2 File), including a number of stretches of 100% identity that are at least 23 nucleotides in length, a proposed limit for successful knockdown [67]. At the same time, a broader comparison (Figure H in S2 File), highlights a general level of approximately 72–83% identity through the VIGS insert region, but with no other regions of 23 or more uninterrupted matches, suggesting knockdown of any of these other genes by the Ta_PYL4AS_A insert is unlikely. However, that the full complement of receptors has yet to be identified in the current draft wheat genome is further confounded by the fact that this work was carried out in cv ‘Fielder’ rather than the sequenced ‘Chinese Spring’ variety. Thus no further attempt was made to decipher the breadth of ABA receptors that were knocked down here.

Further consideration of the receptor knockdown results demonstrates a role for the receptors and ABA signaling pathway in the positive mediation of *F*. *graminearum* infection in wheat heads. That this effect was observed in the absence of any exogenously applied ABA suggests a role for naturally occurring ABA in mediating infection. This is especially interesting considering a recent report that showed that *F*. *graminearum* is able to biosynthesize ABA in its own right [33]. However, whether the source of the ABA stimulating infection is plant- or pathogen-derived, remains to be elucidated. Vice versa, this data also provides evidence that the plant ABA signaling pathway is likely involved in mediating the increase in susceptibility observed previously with the application of exogenous ABA [33, 34].

Interestingly, the effect of BMSV:Ta_PYL4AS_A expression is lost by day 13. While this may be due to ABA only having an effect earlier in infection, the possibilities that the expression of BMSV is reducing over time or that the plant is compensating for reduced ABA receptor expression by increasing the expression of other ABA receptors cannot be strictly eliminated. These aspects remain to be investigated more fully when the full-breadth of ABA receptors in wheat is deciphered. In the interim, with our comparative analog profiling highlighting similarities between wheat and Brachypodium ABA receptors, one might look to the idea of using *B*. *distachyon* as a simplified system to test the relationship between individual ABA receptors and FHB. Indeed it is notable that priming with DON was recently shown to increase FHB resistance in *B*. *distachyon* [68]. While it is not possible to directly reconcile this recent finding with the work reported herein, the possibility that DON is modulating the expression of ABA receptors or other components of the ABA pathway presents an interesting new hypothesis to be tested. Also interesting is that, although the infection level returns to control levels by day 13 after inoculation, the reduction of DON accumulation seems to be propagated for longer. Intriguingly, this suggests that different mechanisms downstream of the ABA-receptors may be mediating FHB infection and DON accumulation respectively. Mechanisms could be linked to hormone cross-talk with other defense related hormones, or could be linked to feedback stimulation of ABA production, where ABA, as a plant secondary metabolite is modulating alternate systems in the plant through interactions with other proteins such as membrane transporters, enzymes involved in ATP synthesis and carbon fixation, and even stress responsive heat shock proteins [69–71]. That there might be multiple distinct pathways and systems mediating these effects is notable being that ABA-receptors are genetically redundant, while at the same time involved in a myriad of other developmental and abiotic responses. Thus while ABA receptors themselves do not present very viable targets for breeding disease resistance, the knowledge that ABA-linked signaling pathways are differentially-linked to responses, suggests that other more practical gene targets for marker development and breeding may eventually be identified, as our understanding of the relationship between ABA and disease responses continues to grow. With respect to the identification of amino acid residue 180 in Ta_PYL2DS_FL as a key modulator of the differential responses of receptors to the ABA-like analog PBI352, it is somewhat unexpected that this residue is located at the interface of the receptor with the PP2C, and not within the ABA binding pocket itself. That the most likely impact of this variation is to weaken the receptor-PP2C interaction is clear from the modelling (Fig 3). However that these two receptors both respond to the high affinity binding of ABA comparably (Fig 2B) suggests that any effect of the weaker receptor-PP2C interaction in D180 containing receptors is only elicited under certain conditions. Further, consideration of other *A*. *thaliana* and *B*. *distachyon* receptors tested here, (Figure C in S2 File) show results consistent with the observation that receptors with ‘D180’ (AtPYR1, Bd37810) show reduced activity compared to those with the ‘E180’ variation (AtPYL5, AtPYL6, Bd32250, Bd09580) (Figure C in S2 File). Similarily, a recent systematic comparison of the interaction of all the *A*.*thaliana* receptors with the same panel of analogs tested herein highlights that the ‘E180’ containing AtPYL5 and AtPYL6 receptors consistently have the highest activity against the PP2Cs, compared to any of the others regardless of the analog tested [38]. That said, there is a degree of predictable variation between all the ‘E180’ containing *A*. *thaliana* receptor responses to individual analogs, likely arising from other amino acid differences that were not tested herein. Lastly, it is interesting to note that previous studies comparing the interaction of *A*. *thaliana* receptors with various PP2Cs in the absence of any ABA, are consistent with E at the position equivalent to E180 in AtPYL5 (as in *A*. *thaliana* PYL4, PYL5, PYL6, PYL8, PYL9, PYL10) mediating higher affinity (stronger responses) for the PP2Cs than those receptors containing Asp at this site (as in *A*. *thaliana* PYR1, PYL1, PYL2 and PYL3) [72]. However that this variation was not detected as activating in a constitutive activation study of AtPYL2 renders this point enigmatic [58].

Furthermore, that there are no differences in the amino acids comprising the ligand binding pockets would suggest that analog affinities for the wheat receptor should be comparable to those detected for AtPYL5 (Figure E in S2 File) [57], although this remains to be confirmed experimentally. However assuming similar affinities, it is interesting to consider a recent isothermal titration calorimetry study that showed that both analogs PBI352 and PBI354 have 30-fold lower affinity for AtPYL5 than ABA [38]. This is reflected herein in the similarity of the responses observed for the *T*. *aestivum* receptors with respect to these two analogs, compared to the response of the *A*. *thaliana* receptors (Fig 2). In contrast analog PBI413 had affinity comparable to ABA, and like ABA shows little difference between receptors. Analog PBI425 had only 6 –fold lower affinity than ABA and shows little variation between receptors here. Finally analog PBI426 has almost 70-fold lower affinity and like PBI352 and PBI354 shows a notable variation between the two receptors. From this we conclude that weaker ligand affinity exacerbates the effect of the weaker PP2C interaction of the ‘D180’ containing receptors. Confirmation of the analog affinities for the wheat receptors, as well as further investigation of the receptor-PP2C binding affinities should shed light on this, but goes beyond the scope of this current study.

Overall *Brachypodium distachyon* and *Triticum aestivum* putative ABA receptors were identified from genomic databases, although identification of the full compliment of wheat ABA receptors awaits the complete genomic sequence. A selection of these were cloned for recombinant expression and their functionality as ABA receptors confirmed by *in vitro* assays against protein phosphatases Type 2Cs, confirming the existence of ABA receptor families in these monocot systems. Mutagenic analyses highlighted Ta_PYL2DS_FL amino acid D180 as a critical contributor to receptor selectivity for different ABA-like analogs. Although it is interesting that the mechanism is linked not to altered ligand specificity, but rather with sensitization of the receptor-PP2C interaction. Finally that knockdown of wheat ABA receptor Ta_PYL4AS_A yielded plants with increased early stage resistance to FHB progression and decreased mycotoxin accumulation, highlights the functional relevance of these receptors and presents novel targets for investigation in the future development of disease resistant crops.

## Supporting Information

S1 File Table A in S1 File. Locator ID’s and associated sequences of *A*. *thaliana*, *B*. *distachyon* and *T*. *aestivum* genes and proteins identified and characterized in this work. Locators are NCBI Gene Identifiers whenever available. For wheat sequences where GI numbers are not available the identifier refers to the IWGSC sequence survey database, for contigs_longerthan_200 for each chromosome.
(XLSX)

S2 File Figures A-H in S2 File. (DOCX)

## References

[1] Wasilewska A, Vlad F, Sirichandra C, Redko Y, Jammes F, Valon C, et al An update on abscisic acid signaling in plants and more. Mol Plant. 2008;1: 198–217. 10.1093/mp/ssm022 19825533

[2] Ma Y, Szostkiewicz I, Korte A, Moes D, Yang Y, Christmann A, et al Regulators of PP2C phosphatase activity function as abscisic acid sensors. Science. 2009;324: 1064–1068. 10.1126/science.1172408 19407143

[3] Park SY, Fung P, Nishimura N, Jensen DR, Fujii H, Zhao Y, et al Abscisic acid inhibits type 2C protein phosphatases via the PYR/PYL family of START proteins. Science. 2009;324: 1068–1071. 10.1126/science.1173041 19407142 PMC2827199

[4] Nishimura N, Hitomi K, Arvai AS, Rambo RP, Hitomi C, Cutler SR, et al Structural mechanism of abscisic acid binding and signaling by dimeric PYR1. Science. 2009;326: 1373–1379. 10.1126/science.1181829 19933100 PMC2835493

[5] Santiago J, Dupeux F, Round A, Antoni R, Park SY, Jamin M, et al The abscisic acid receptor PYR1 in complexwith abscisic acid. Nature. 2009a;462: 665–668. 19898494 10.1038/nature08591

[6] Miyazono K, Miyakawa T, Sawano Y, Kubota K, Kang H, Asano A, et al Structural basis of abscisic acid signalling. Nature. 2009;462: 609–614. 10.1038/nature08583 19855379

[7] Yin P, Fan H, Hao Q, Yuan X, Wu D, Pang Y, et al Structural insights into the mechanism of abscisic acid signaling by PYL proteins. Nat Struct Mol Biol. 2009;16:1230–1236. 10.1038/nsmb.1730 19893533

[8] Melcher K, Ng LM, Zhou XE, Soon FF, Xu Y, Suino-Powell KM, et al A gate-latch-lock mechanism for hormone signalling by abscisic acid receptors. Nature. 2009;462: 602–608. 10.1038/nature08613 19898420 PMC2810868

[9] Raghavendra AS, Gonugunta VK, Christmann A, Grill E. ABA preception and signaling. Trends Plant Sci. 2010;15: 395–401. 10.1016/j.tplants.2010.04.006 20493758

[10] Cutler SR, Rodriguez PL, Finkelstein RR, and Abrams SR. Abscisic acid: emergence of a core signaling network. Annu Rev Plant Biol. 2010;61: 651–679. 10.1146/annurev-arplant-042809-112122 20192755

[11] Chai YM, Jia HF, Li CL, Dong QH, Shen YY. FaPYR1 is involved in strawberry fruit ripening. J Exp Bot. 2012;62: 5079–5089. 10.1093/jxb/err207 21778181

[12] Jia HF, Chai YM, Li CL, Lu D, Luo JJ, Qin L, et al Abscisic acid plays an important role in the regulation of strawberry fruit ripening. Plant Physiol. 2011;157: 188–199. 10.1104/pp.111.177311 21734113 PMC3165869

[13] Li C, Jia H, Chai Y, Shen Y. Abscisic acid perception and signaling transduction in strawberry: a model for non-climacteric fruit ripening. Plant Signal Behav. 2011;6: 1950–1953. 10.4161/psb.6.12.18024 22095148 PMC3337185

[14] Boneh U, Biton I, Zheng C, Schwartz A, Ben-Ari G. Characterization of potential ABA receptors in Vitis vinifera. Plant Cell Rep. 2012;31: 311–321. 10.1007/s00299-011-1166-z 22016084

[15] Li G, Xin H, Zheng XF, Li S, Hu Z, Identification of the abscisic acid receptor VvPYL1 in Vitis vinifera. Plant Biol (Stuttg). 2012;14: 244–248. 21974741 10.1111/j.1438-8677.2011.00504.x

[16] Romero P, Lafuente MT, Rodrigo MJ. The citrus ABA signalosome: identification and transcriptional regulation during sweet orange fruit ripening and leaf dehydration. J Exp Bot. 2012;63: 4931–4945. 10.1093/jxb/ers168 22888124 PMC3428003

[17] Wang Y, Wu Y, Duan C, Chen P, Li Q, Dai S, et al The expression profiling of the Cs PYL, CsPP2C and CsSnRK2 gene families during frout development and drought stress in cucumber. J Plant Physiol. 2012;169: 1874–1882. 10.1016/j.jplph.2012.07.017 22959675

[18] Bai G, Yang DH, Zhao Y, Ha S, Yang F, Ma J, et al Interactions between soybean ABA receptors and type 2C protein phosphatases. Plant Mol Biol. 2013;83: 651–664. 10.1007/s11103-013-0114-4 23934343 PMC3834219

[19] Kim H, Hwang H, Hong JW, Lee YN, Ahn IP, Yoon IS, et al A rice orthologue of the ABA receptor, OsPYL/RCAR5, is a positive regulator of the ABA signal transduction pathway in seed germination and early seedling growth. J Exp Bot. 2012;63: 1013–1024. 10.1093/jxb/err338 22071266

[20] He Y, Hao Q, Li W, Yan C, Yan N, Yin P. Identification and characterization of ABA receptors in Oryza sativa. PLoS One 2014;9: e95246 10.1371/journal.pone.0095246 24743650 PMC3990689

[21] Seiler C, Harshavardhan VT, Reddy PS, Hensel G, Kumlehn J, Eschen-Lippold L, et al Abscisic Acid Signaling Responses and Impact Assimilation Efficiency in Barley under Terinal Drought Stress. Plant Physiol. 2014;164: 1677–1696. 10.1104/pp.113.229062 24610749 PMC3982733

[22] Tian X, Wang Z, Li X, Lv T, Liu H, Wang L, et al Characterization and Functional Analysis of Pyrabactin Resistance-Like Abscisic Acid Receptor Family in Rice. Rice. 2015;8: 28 10.1186/s12284-015-0061-6 26362328 PMC4567572

[23] Mauch-Mani B, Mauch F. The role of abscisic acid in plant-pathogen interactions. Curr Opin Plant Biol. 2008;8: 409–14. 10.1016/j.pbi.2005.05.015 15939661

[24] Asselbergh B, De Vleesschauwer D, Hofte M. Global switches and fine-tuning- ABA modulates plant pathogen defense. Mol Plant Microbe Interact. 2008;21:709–19. 10.1094/MPMI-21-6-0709 18624635

[25] Robert-Seilaniantz A, Grant M, Jones JDG. Hormone crosstalk in plant disease and defense: More than just JASMONATE-SALICYLATE antagonism. Annu Rev Phytopathol. 2011;49: 317–43. 10.1146/annurev-phyto-073009-114447 21663438

[26] Cao FY, Yoshioka K, and Desveaux D. The roles of ABA in plant-pathogen interactions. J Plant Res. 2011;124: 489–499. 10.1007/s10265-011-0409-y 21380629

[27] Lim CW, Luan S, Lee SC. A Prominent Role for RCAR3-Mediated ABA Signaling in Response to Pseudomonas syringae pv. tomato DC3000 Infection in Arabidopsis. Plant and Cell Physiol. 2014;55: 1691–1703. 25063782 10.1093/pcp/pcu100

[28] Anderson JP, Badruzsaufari E, Schenk PM, Manners JM, Desmond OJ, Ehlert C, et al Antagonistic interaction between abscisic acid and jasmonate-ethylene signaling pathways modulates defense gene expression and disease resistance in Arabidopsis. Plant Cell 2004;16: 3460–3479. 10.1105/tpc.104.025833 15548743 PMC535886

[29] Trusov Y, Sewelam N, Rookes JE, Kunkel M, Nowak E, Schenk PM, et al Heterotrimeric G-proteins-mediated resistance to necrotrophic pathogens includes mechanisms independent of salicylic acid-, jasmonic acid/ethylene- and abscisic acid-mediated defense signaling. Plant J. 2009;58: 69–81. 10.1111/j.1365-313X.2008.03755.x 19054360

[30] Sanchez-Vallet A, Lopez G, Ramos B, Delgado-Cerezo M, Riviere MP, Lorente F, et al Disruption of abscisic acid signaling constitutively activates Arabidopsis resistance to the necrotrophic fungus Plectosphaerella cucumerina. Plant Physiol. 2012:160; 2109–2124. 10.1104/pp.112.200154 23037505 PMC3510135

[31] Koga H, Dohi K, Mori M. Abscisic acid and low temperatures suppress the whole plant-specific resistance reaction of rice plants to the infection of Magnaporthe grisea. Physiol Mol Plant Pathol. 2004;65: 3–9.

[32] Ulferts S, Delventhal R, Splivallo R, Karlovsky P, Schaffrath U. Abscisic acid negatively interferes with basal defense of barley against Magnaporthe oryzae. BMC Plant Biol. 2015; 10.1186/s12870-014-0409-x 25604965 PMC4307682

[33] Qi P-F, Balcerzak M, Rocheleau H, Leung W, Wei Y-M, Zheng Y-L, et al Jasmonic acid and abscisic acid play important roles in host-pathogen interaction between Fusarium graminearum and wheat during the early infection stages of Fusarium head blight. Physiol Mol Plant Pathol. 2016;93: 39–48.

[34] Buhrow LM, Cram D, Tulpan D, Foroud NA, Loewen MC. Exogenous abscisic acid and gibberellic acid elicit opposing effects on Fusarium graminearum infection in wheat. Phytopathology. 2016a; 10.1094/PHYTO-01-16-0033-R 27135677

[35] McMullen M, Jones R, and Gallenberg D. Scab of wheat and barley: a re-emerging disease of devastating impact. Plant Dis. 1997;81: 1340–1348. 10.1094/PDIS.1997.81.12.1340 30861784

[36] McCormick S. The role of DON in pathogenicity In: Leonard K. J., Bushnell W. R., editors. Fusarium Head Blight of Wheat and Barley. American Phytopathological Society; St. Paul, MN, USA: 2003: pp. 165–183.

[37] Kang Z, and Buchenauer H. Immunocytochemical localization of fusarium toxins in infected wheat spikes by Fusarium culmorum. Physiol Mol Plant Pathol. 1999;55: 275–288.

[38] Boratyn GM, Camacho C, Cooper PS, Coulouris G, Fong A, Ma N, et al BLAST: a more efficient report with usability improvements. Nucl Acids Res. 2013;41: W29–W33. 10.1093/nar/gkt282 23609542 PMC3692093

[39] Sievers F, Wilm A, Dineen D, Gibson TJ, Karplus K, Li W, et al Fast, scalable generation of high-qality protein multiple sequenc alignemnts using clustal Omega. Mol Sys Biol. 2011;7: 539. 10.1038/msb.2011.75 PMC3261699 21988835

[40] Tamura K, Stecher G, Peterson D, Filipski A, and Kumar S. MEGA6: Molecular Evolutionary Genetics Analysis version 6.0. Mol Biol Evol. 2013;30: 2725–2729. 10.1093/molbev/mst197 24132122 PMC3840312

[41] Nakamura S, Komatsuda T, Miura H. Mapping diploid wheat homologues of Arabidopsis seed ABA signaling genes and QTLs for seed dormancy. Theor Appl Genet. 2007;114: 1129–1139. 10.1007/s00122-007-0502-8 17387417

[42] Benson CL, Kepka M, Wunschel C, Rajagopalan N, Nelson KM, Christmann A, et al Abscisic acid analogs as chemical probes for dissection of abscisic acid responses in Arabidopsis thaliana. Phytochemistry. 2015;113: 96–107. 10.1016/j.phytochem.2014.03.017 24726371

[43] Bradford MM. A rapid and sensitive method for the quantitation of microgram quantities of protein utilizing the principle of protein-dye binding. Anal Biochem. 1976;72: 248–254. 942051 10.1016/0003-2697(76)90527-3

[44] Proctor RH, Hohn TM, McCormick SP. Reduced virulence of Gibberella zeae caused by disruption of a tricholthecene toxin biosynthesis gene. Mol Plant Microbe Interact. 1995;8: 593–601. 8589414 10.1094/mpmi-8-0593

[45] Buhrow LM, Clark SM, Loewen MC. Identification of an attenuated barley stripe mosaic virus for the virus-induced gene silencing of pathogenesis-related wheat genes. Plant Methods. 2016b; 12:12 10.1186/s13007-016-0112-z 26839581 PMC4736275

[46] Koressaar T, Remm M. Enhancements and modifications of primer design program Primer3. Bioinformatics. 2007;23: 1289–1291. 10.1093/bioinformatics/btm091 17379693

[47] Untergasser A, Cutcutache I, Koressaar T, Ye J, Faircloth BC, Remm M, and Rozen SG. Primer3—new capabilities and interfaces. Nucleic Acids Res. 2012;40: e115 10.1093/nar/gks596 22730293 PMC3424584

[48] Yuan C, Li C, Yan L, Jackson AO, Liu Z, Han C, et al A high throughput barley stripe mosaic virus vector for virus induced gene silencing in monocots and dicots. PLoS One. 2011;6: e26468 10.1371/journal.pone.0026468 22031834 PMC3198768

[49] Sambrook J, Fritschi EF and Maniatis T. Molecular cloning: a laboratory manual, Cold Spring Harbor Laboratory Press, New York 1989.

[50] Plattner RD, Maragos CM. Determination of deoxynivalenol and nivalenol in corn and wheat by liquid chromatography with electrospray mass spectrometry. J AOAC Int. 2003;86; 61–65. 12607741

[51] International Wheat Genome Sequencing. A chromosome-based draft sequence of the hexaploid bread wheat (Triticum aestivum) genome. Science. 2012;345: 1251788. 10.1126/science.1251788 25035500

[52] Szostkiewicz I, Richter K, Kepka M, Demmel S, Ma Y, Korte A, et al Closely related receptor complexes differ in their ABA selectivity and sensitivity. Plant J. 2010;61: 25–35. 10.1111/j.1365-313X.2009.04025.x 19769575

[53] Saitou N, and Nei M. The neighbor-joining method: A new method for reconstructing phylogenetic trees. Mol Biol Evol. 1987;4: 406–425. 3447015 10.1093/oxfordjournals.molbev.a040454

[54] Felsenstein J. Confidence limits on phylogenies: An approach using the bootstrap. Evolution. 1985;39: 783–791. 28561359 10.1111/j.1558-5646.1985.tb00420.x

[55] Zuckerkandl E, and Pauling L. Evolutionary divergence and convergence in proteins Edited in Evolving Genes and Proteins by Bryson V. and Vogel H.J., Academic Press, New York 1965 pp. 97–166.

[56] Dupeux F, Santiago J, Betz K, Twycross J, Gonzalez-Guxman M, Jensen MR, Rodriguez PL, Marques JA, et al A thermodynamic switch modulates abscisic acid receptor sensitivity. EMBO J. 2011;30: 4171–4184. 10.1038/emboj.2011.294 21847091 PMC3199383

[57] Dorosh l, Kharenko OA, Rajagopalan N, Loewen MC, Stepanova M. Molecular mechanisms in the activation of abscisic acid receptor PYR1. PLoS Comput Biol. 2013;9:e1003114 10.1371/journal.pcbi.1003114 23825939 PMC3694813

[58] Mosquna A, Peterson FC, Park S-Y, Lozano-Juste J, Volkman BF, Cutler SR. Potent and selective activation of abscisic acid receptors in vivo by mutational stabilization of their agonist-bound conformation. Proc Natl Acad Sci USA. 2011;108: 20838–20843. 10.1073/pnas.1112838108 22139369 PMC3251050

[59] Biasini M, Bienert S, Waterhouse A, Arnold K, Studer G, Schmidt T, et al SWISS-MODEL: modelling protein tertiary and quaternary structure using evolutionary information. Nucleic Acids Res. 2014; 42: W252–W258. 10.1093/nar/gku340 24782522 PMC4086089

[60] Gonzalez-Guzman M, Pizzio GA, Antoni R, Vera-Sirera F, Marilo E, Bassel GW, et al Arabidopsis PYR/PYL/RCAR receptors play a major role in quantitative regulation of stomatal aperture and transcriptional response to abscisic acid. Plant Cell. 2012:24: 2483–2496. 10.1105/tpc.112.098574 22739828 PMC3406898

[61] Geiger D, Maierhofer T, Al-Rasheid KA, Scherzer S, Mumm P, Liese A, et al Stomatal closure by fast abscisic acid signaling is mediated by the guard cell anion channel SLAH3 and the receptor RCAR1. Sci Signal. 2011;4, ra32 10.1126/scisignal.2001346 21586729

[62] Lackman P, Gonzalez-Guzman M, Tilleman S, Carqueijeiro I, Perez AC, Moses T, et al Jasmonate signaling involves the abscisic acid receptor PYL4 to regulate metabolic reprogramming in Arabidopsis and tobacco. Proc Natl Acad Sci USA. 2011;108: 5891–5896. 10.1073/pnas.1103010108 21436041 PMC3078376

[63] Santiago J, Rodrigues A, Saez A, Rubio S, Antoni R, Dupeux F, et al Modulation of drought resistance by the abscisic acid receptor PYL5 through inhibition of clade A PP2Cs. Plant J. 2009b;60: 575–588. 19624469 10.1111/j.1365-313X.2009.03981.x

[64] Pizzio GA, Rodriguez L, Antoni R, Gonzalez-Guzman M, Yunta C, Merilo E, et al The PYL4 A194T mutant uncovers a key role ofr PYL4/PROTEIN PHOSPHATASE 2CA interaction for abscisic acid signaling and plant drought resistance. Plant Physiol. 2013;163: 441–455. 10.1104/pp.113.224162 23864556 PMC3762663

[65] Antoni R, Gonzalez-Guzman M, Rodriguez L, Peirats-Llobet M, Pizzio GA, Fernandez MA, et al PYL8 plays an important role for regulation of abscisic acid signaling in root. Plant Physiol. 2013;161: 931–941. 10.1104/pp.112.208678 23370718 PMC3561030

[66] Okamoto M, Peterson FC, Defries A, Park S-Y, Endo A, Nambara E, et al Activation of dimeric ABA receptor5s elicits guard cell closure, ABA-regulated gene expression and drought tolerance. Proc Natl Acad Sci USA. 2013;110: 12132–37. 10.1073/pnas.1305919110 23818638 PMC3718107

[67] Thomas CL, Jones L, Baulcombe DC, Maule AJ. Size constraints for targeting post-transcriptional gene silencing and for RNA-directed methylation in Nicotiana benthamiana using a potato virus X vector. Plant J. 2001;25: 417–25. 11260498 10.1046/j.1365-313x.2001.00976.x

[68] Blumke A, Sode B, Ellinger D, Voigt CA. Reduced Susceptibilty to Fusarium head blight in Brachypodium distachyon through priming with the Fusarium mycotoxin deoxynivalenol. Mol Plant Pathol. 2015;16: 472–483. 10.1111/mpp.12203 25202860 PMC6638442

[69] Kharenko OA, Boyd J, Nelson KM, Abrams SR, Loewen MC. Identification and characterization of interactions between abscisic acid and mitochondrial adenine nucleotide translocators. Biochem J. 2011;437:117–23. 10.1042/BJ20101898 21473740

[70] Kharenko OA, Polichuk D, Nelson KM, Abrams SR, Loewen MC. Identification and characterization of interactions between abscisic acid and human heat shock protein 70 family members. J Biochem. 2013; 154: 383–91. 10.1093/jb/mvt067 23975754

[71] Galka MM, Rajagopalan N, Buhrow LM, Nelson KM, Switala J, Cutler AJ, et al Identification of Interactions between Abscisic Acid and Ribulose-1,5-Bisphosphate Carboxylase/Oxygenase. PLoS One. 2015;10: e0133033 10.1371/journal.pone.0133033 26197050 PMC4510133

[72] Hao Q, Yin P, Li W, Wang L, Yan C, Lin Z, et al The molecular basis of ABA-independent inhibition of PP2Cs by a subclass of PYL Proteins. Mol Cell. 2011;42: 662–672. 10.1016/j.molcel.2011.05.011 21658606

