# Supplementary material for: Characterization of Triticum aestivum Abscisic Acid Receptors and a Possible Role for These in Mediating Fusairum Head Blight Susceptibility in Wheat
Source: PLoS One. 2016 Oct 18;11(10):e0164996. doi: 10.1371/journal.pone.0164996 (PMC5068739; doi:10.1371/journal.pone.0164996)
Supplement: S2 File — (DOCX) [file pone.0164996.s002.docx]

**SUPPLEMENTAL FIGURES for Gordon et al.**

**Characterization of *Triticum aestivum* abscisic acid receptors and a possible role for these in mediating *Fusarium* head blight susceptibility in wheat**

**Cameron S. Gordon^1,2^, Nandhakishore Rajagopalan^2^, Eddy P. Risseeuw^2^, Marci Surpin^3^, Fraser J. Ball^2^, Carla J. Barber^2^, Leann M. Buhrow^2^, Shawn M. Clark^2^, Jonathan E. Page^2^, Chris D. Todd^4^, Suzanne R. Abrams^2,5^ , Michele C. Loewen^1,2^***

^1^ Department of Biochemistry, University of Saskatchewan, 107 Wiggins Rd., Saskatoon, SK, S7N 5E5, Canada

^2^ National Research Council of Canada, 110 Gymnasium Place, Saskatoon, SK, S7N 0W9, Canada

^3^ Valent BioSciences Corporation, 870 Technology Way, Libertyville, Illinois 60048 USA

^4^ Department of Biology, University of Saskatchewan, 112 Science Place, Saskatoon, SK, S7N 5E2, Canada

^5^ Department of Chemistry, University of Saskatchewan, 110 Science Place, Saskatoon, SK, S7N 5C9, Canada

Corresponding Author: michele.loewen@nrc.ca


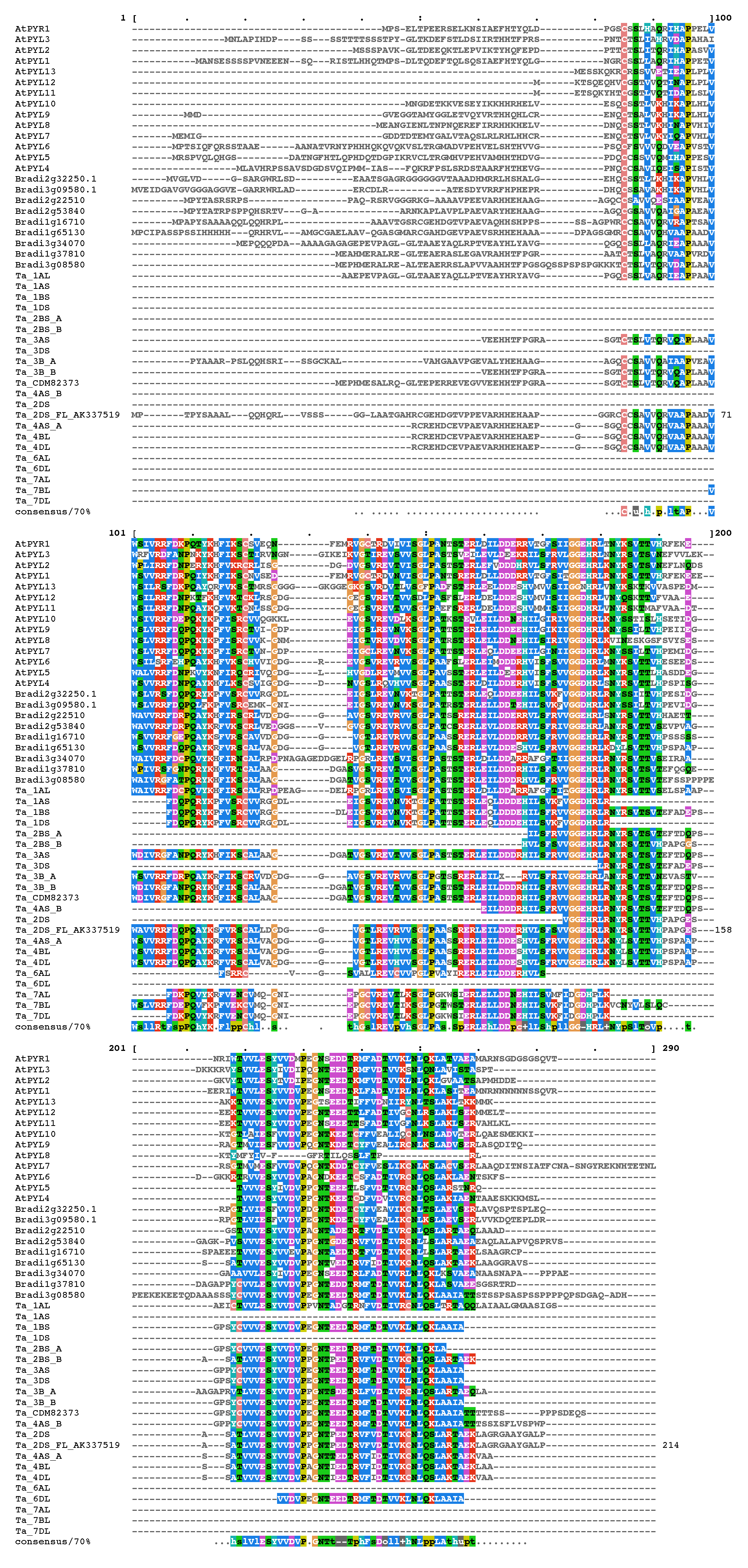


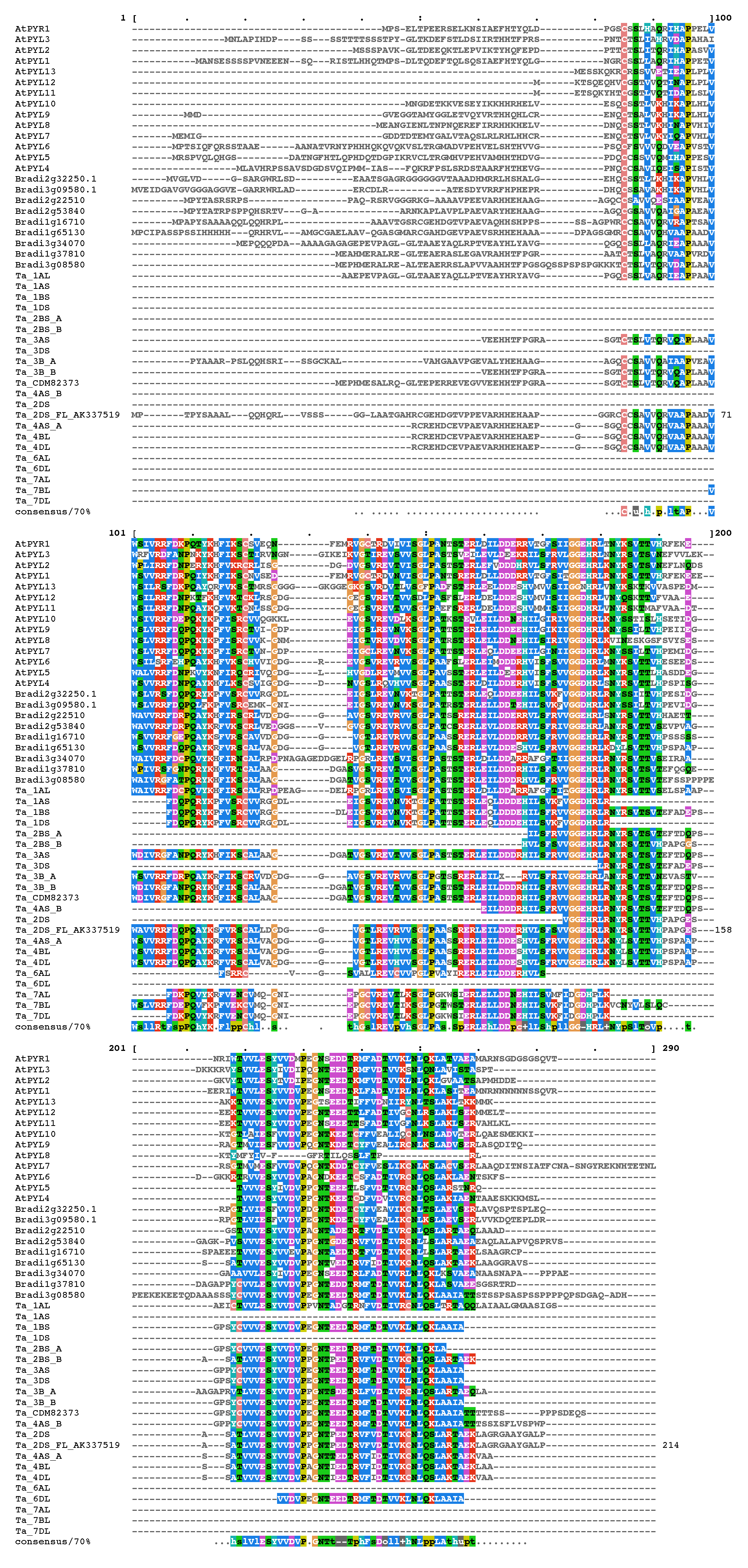


**Figure A: Amino acid Alignement of *T. aestivum* and *B. distachion* putative ABA receptors with *A. thaliana* ABA receptors.** The alignment was performed using Clustal Omega (Sievers et al., 2011).


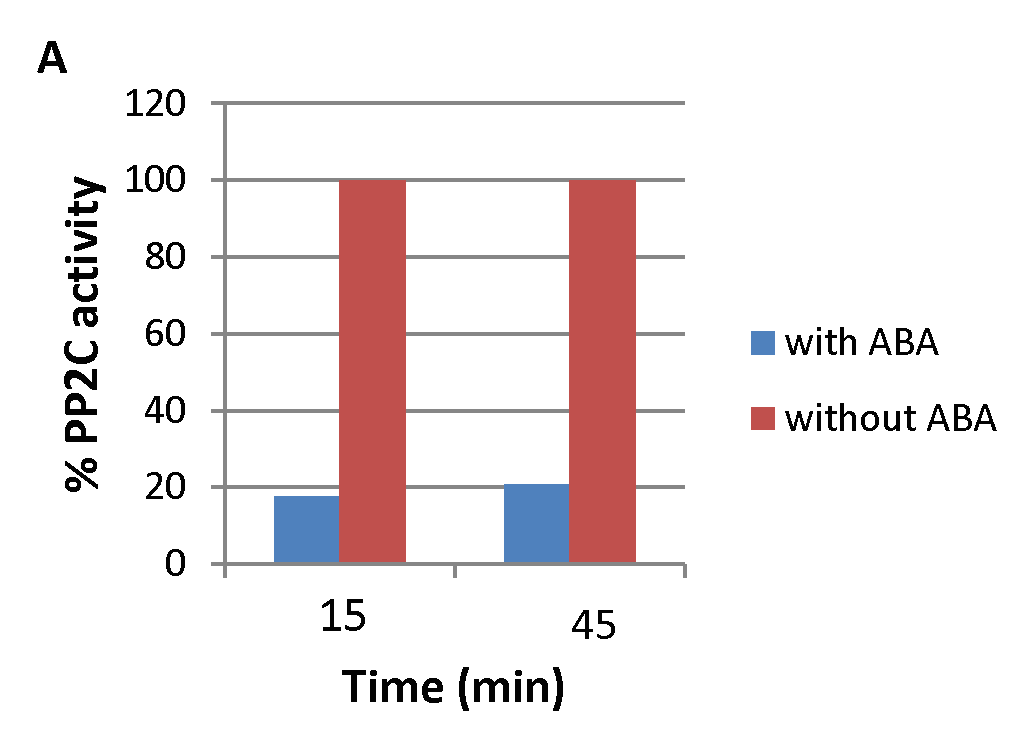


**Figure B: Ta_2DF_FL selectively inhibits PP2Cs in an ABA-dependent manner.** The activity of recombinantly produced and enriched wheat TaABI1 PP2C was assessed in the presence of the Ta_2DS_FL ABA receptor at two different time points, in the presence and absence of 10 μM ABA. The protein phosphatase activity was analyzed at a constant molar ratio of receptor to PP2C of 2:1 *in vitro*.

**Figure C:** **ABA Analog Activity Profiles are Dependent on the Identity of the Receptor.** The modulation of the activity of the *B. distachyon* Bradi2g41950 PP2C (homologue of AtABI1) was assessed in the presence of three different *B. distachyon* receptors as indicated, against various enantiomeric ABA analog pairs (n=1). The modulation of the activity of the wheat TaABI1 PP2C was assessed in the presence of three different *A. thaliana* receptors, AtPYR5, AtPYR6 and PtPYR1, against various enantiomeric ABA analog pairs (with standard error show by the error bars, n=3). Top: (+)-enanatiomers, Bottom: (-)-enantomers. Roman numerals in the legend indicate phylogenetic subfamily. The protein phosphatase activity was analyzed at a constant molar ratio of receptor to PP2C of 2:1 *in vitro* at a constant analog concentration of 1.0 μM.

**Figure D:** **ABA Analog Activity Profiles are Less Dependent on the Identity of the PP2C.** The modulation of the activities of three *A. thaliana* and one wheat PP2Cs were assessed in the presence of the wheat Ta_2DS_FL receptor, against various enantiomeric ABA analog pairs. Top: (+)-enanatiomers, Bottom: (-)-enantomers. The protein phosphatase activity was analyzed at a constant molar ratio of receptor to PP2C of 2:1 *in vitro* at a constant analog concentration of 1.0 μM (n=3).

TA_2DS_FL MPTPYSAAALQQHQRLVSSSGGLAATGA--------------HRCGEHDGTVPPEVARHH 46

AtPYR1 MRSPVQ-----------LQHGSDATNGFHTLQPHDQTDGPIKRVCLTRGMHVPEHVAMHH 49

Conservation * :* . . *. *:.* : * : ** .** **

DOROSH – ABA

DOROSH – PP2C

TA_2DS_FL EHAAPGGRCCCSAVVQRVAAPAADVWAVVRRFDQPQAYKSFVRSCALLDGDG-GVGTLRE 105

AtPYR1 TH-DVGPDQCCSSVVQMIHAPPESVWALVRRFDNPKVYKNFIRQCRIVQGDGLHVGDLRE 108

Conservation * * ***:*** : ** .***:*****:*:.**.*:*.* :::*** ** ***

DOROSH – ABA # #

DOROSH – PP2C ## #

TA_2DS_FL VRVVSGLPAASSRERLEILDDERHVLSFSVVGGEHRLRNYRSVTTVHPAPGESASATLVV 165

AtPYR1 VMVVSGLPAVSSTERLEILDEERHVISFSVVGGDHRLKNYRSVTTLHASDD---EGTVVV 165

Conservation * *******.** *******:****:*******:***:*******:* : ..*:**

DOROSH – ABA # ### # # # # ### # #

DOROSH – PP2C ##### ##

TA_2DS_FL ESYVVDVPPGNTPEDTRVFVDTIVKCNLQSLARTAEKLAGRGAAYGALP 214

AtPYR1 ESYIVDVPPGNTEEETLSFVDTIVRCNLQSLARSTNRQ----------- 203

Conservation ***:******** *:* ******:********::::

DOROSH – ABA # ## ## ##

DOROSH – PP2C # # # ## # #

**Figure E: Amino acid alignment of Ta_2DS_FL with AtPYL5.** Highlighted by hashtags under the alignment are amino acid sites predicted to contribute to the Receptor-ABA interaction (Dorosh – ABA) and the Receptor-PP2C interaction (Dorosh – PP2C) as predicted in Dorosh et al 2013. Of the 33 sites predicted to be involved in interactions, the three sites that vary between the two receptors are highlighted in yellow. In wheat these residues include S86, D180 and V183, in *A. thaliana* the same sites are N88, E180 and S183. The alignment was made using Clustal Omega (Sievers et al., 2011). An ‘*’ (asterisk) indicates positions which have a single, fully conserved residue. A ‘:’ (colon) indicates conservation between groups of strongly similar properties - scoring > 0.5 in the Gonnet PAM 250 matrix.  A ‘.’ (period) indicates conservation between groups of weakly similar properties - scoring =< 0.5 in the Gonnet PAM 250 matrix.

**A**

TA_4AS_A AGGTGCAGGGAGCACGACTGCGAGGTCCCGGCGGAGGTGGCGCGGCACCACGAGCACGCG 60

VIGS_TA_4AS_A ------------------------------------------------------------ 0

VIGS_TA_2DS_FL ------------------------------------------------------------ 0

TA_4AS_A GAGCCGGGGTCCGGCCAGTGCTGCTCCGCGGTGGTGCAGCACGTGGCGGCGCCCGCGGCG 120

VIGS_TA_4AS_A ------------------------------------------------------------ 0

VIGS_TA_2DS_FL ------------------------------------------------------------ 0

TA_4AS_A GCGGTGTGGTCCGTGGTGCGCCGGTTCGACCAGCCGCAGGCGTACAAGCGGTTCGTCCGC 180

VIGS_TA_4AS_A ------------------------------------------------------------ 0

VIGS_TA_2DS_FL ------------------------------------------------------------ 0

TA_4AS_A AGCTGCGCCCTGGTGGCCGGTGACGGCGGCGTGGGCACGCTCCGCGAGGTGCACGTCGTG 240

VIGS_TA_4AS_A ------------------------------------------------------------ 0

VIGS_TA_2DS_FL ------------------------------------------------------------ 0

TA_4AS_A TCGGGCCTCCCCGCGGCGTCCAGCCGCGAGCGGCTCGAGATCCTGGACGACGAGAGCCAC 300

VIGS_TA_4AS_A --------------------------------GTCGGAGATCCTGGACGACGAGAGCCAC 28

VIGS_TA_2DS_FL --------------------------------GCTGGAGATCCTGGACGACGAGCGGCAC 28

Conservation * ****************** * ***

TA_4AS_A GTGCTCAGCTTCCGCGTCGTCGGCGGGGAGCACCGGCTCAAGAACTACCTCTCCGTCACC 360

VIGS_TA_4AS_A GTGCTCAGCTTCCGCGTCGTCGGCGGGGAGCACCGGCTCAAGAACTACCTCTCCGTCACC 88

VIGS_TA_2DS_FL GTGCTGAGCTTCAGCGTGGTGGGCGGCGAGCACCGGCTCCGCAACTACCGGTCGGTGACC 88

Conservation ***** ****** **** ** ***** ************ ******* ** ** ***

TA_4AS_A ACCGTGCACCCATCCCCGGCCGC--GCCGTCCAGCGCCACCGTCGTCGTGGAGTCGTACG 418

VIGS_TA_4AS_A ACCGTGCACCCATCCCCGGCCGCGC--CGTCCAGCGCCACCGTCGTCGTGGAGTCGTACG 146

VIGS_TA_2DS_FL ACGGTGCACCCGGCGCCGGGGGAGAGCGCGTCGGCGACGC--TGGTGGTGGAGTCGTACG 146

Conservation ** ******** * **** * * *** * * * ** *************

TA_4AS_A TCGTGGACGTGCCCGCGGGCAACACGACCGAGGACACCCGCGTGTTCATCGACACCATCG 478

VIGS_TA_4AS_A TCGTGGACGTGCCCGCGGGCAACACGACCGAGGACACCCGCGTGTTCATCGACACCATCG 206

VIGS_TA_2DS_FL TGGTGGACGTGCCCCCCGGGAACACGCCCGAGGACACCCGCGTCTTCGTGGACACCATCG 206

Conservation * ************ * ** ****** **************** *** * **********

TA_4AS_A TCAAGTGCAACCTCCAGTCGCTGGCCAAGACCGCCGAGAAGGTCGCCGCC 528

VIGS_TA_4AS_A TCAAGTGCAAC--------------------------------------- 217

VIGS_TA_2DS_FL TCAAGTGCAAC--------------------------------------- 217

Conservation ***********

**B**

1: TA_4AS_A 100.00 98.60 81.86

2: VIGS_TA_4AS_A 98.60 100.00 80.93

3: VIGS_TA_2DS_FL 81.86 80.93 100.00

**C**

>GFP

GACGACGGCAACTACAAGACCCGCGCCGAGGTGAAGTTCGAGGGCGACACCCTGGTGAACCGCATCGAGCTGAAGGGCATCGACTTCAAGGAGGACGGCAACATCCTGGGGCACAAGCTGGAGTACAACTACAACAGCCACAACGTCTATATCATGGTCGACAAGCAGAAGAACGGCATCAAGGTGAACTTCAAGATCCGCCAC

**Figure F. Comparison of the planned VIGS Ta_2DS_FL VIGS insert to the the amplified Ta_4AS_A VIGS cDNA insert.** Alignment of the cDNA insert regions for Ta_4AS_A that was amplified from from *T. aestivum* cv ‘Fielder’ compared to the insert that was planned based on primers (yellow) designed to amplify from the *T. aestivum* cv ‘Chinese Spring’ Ta_2DS_FL cDNA. B) Sequence identity matrix comparing amplified VIGS_Ta_4AS_A fragement to that of Ta_4AS_A and the planned VIGS_Ta_2DS_FL insert. Alignments and identities obtained using Clustal Omega (Sievers et al., 2011). An ‘*’ (asterisk) indicates positions which have a single, fully conserved residue. C) cDNA sequence of the GFP VIGS insert used in Control experiments.

**A**

VIGS_TA_4AS_A GTCGGAGATCCTGGACGACGAGAGCCACGTGCTCAGCTTCCGCGTCGTCGGCGGGGAGCA 60

TA_4BL GCTCGAGATCCTGGACGACGAGAGCCACGTGCTCAGCTTCCGCGTCGTCGGTGGCGAGCA 60

TA_4DL GCTCGAGATCCTGGACGACGAGAGCCACGTGCTCAGTTTCCGCGTCGTCGGTGGCGAGCA 60

Conservation * ******************************** ************** ** *****

VIGS_TA_4AS_A CCGGCTCAAGAACTACCTCTCCGTCACCACCGTGCACCCATCCCCGGCCGCGCCGTCCAG 120

TA_4BL CCGGCTCAAGAACTACCTCTCCGTCACCACCGTCCACCCGTCCCCGGCCGCGCCGTCCAG 120

TA_4DL CCGGCTCAAGAACTACCTCTCCGTCACCACCGTCCACCCGTCCCCAGCCGCGCCGTCCAG 120

Conservation ********************************* ***** ***** **************

VIGS_TA_4AS_A CGCCACCGTCGTCGTGGAGTCGTACGTCGTGGACGTGCCCGCGGGCAACACGACCGAGGA 180

TA_4BL CGCCACCGTCGTCGTGGAGTCGTACGTCGTGGACGTGCCGGCGGGCAACACGATCGAGGA 180

TA_4DL CGCCACCGTCGTCGTGGAGTCCTACGTCGTGGACGTGCCGGCGGGCAACACGATCGAGGA 180

Conservation ********************* ***************** ************* ******

VIGS_TA_4AS_A CACCCGCGTGTTCATCGACACCATCGTCAAGTGCAAC 217

TA_4BL CACCCGCGTGTTCATCGACACCATCGTCAAGTGCAAC 217

TA_4DL CACCCGCGTGTTCATCGACACCATCGTCAAGTGCAAC 217

Conservation *************************************

**B**

1: VIGS_TA_4AS_A 100.00 95.85 94.47

2: TA_4BL 95.85 100.00 98.62

3: TA_4DL 94.47 98.62 100.00

**Figure G. Evaluation of nucleotide polymorphisms in the two closest homologs of the amplified Ta_4AS_A VIGS cDNA insert.** A) cDNA alignment of the cDNA insert regions for Ta_4AS_A against Ta_4BL and Ta_4DL. B) Sequence identity matrix comparing Ta_4AS_A to Ta_BL and Ta_DL for cDNA encoding the VIGS insert region. Alignments and identities obtained using Clustal Omega (Sievers et al., 2011). An ‘*’ (asterisk) indicates positions which have a single, fully conserved residue.

**A**

TA_3B_A GCTCGAGATTCTGG---------NGCGGGTGCTCAGCTTCCGGATCGTGGGCGGTGAGCA 51

TA_2DS_FL GCTGGAGATCCTGGACGACGAGCGGCACGTGCTGAGCTTCAGCGTGGTGGGCGGCGAGCA 60

TA_3AS CCTCGAGATCCTCGACGACGACCGCCACATCCTCAGCTTCCGCGTCGTCGGCGGCGAGCA 60

TA_CDM82373 CCTCGAGATCCTCGACGACGACCGCCACATCCTCAGCTTCCGCGTCGTCGGCGGCGAGCA 60

TA_3B_B CCTCGAGATCCTCGACGACGACCGCCACATCCTCAGCTTCCGCGTCGTCGGCGGCGAGCA 60

VIGS_TA_4AS_A GTCGGAGATCCTGGACGACGAGAGCCACGTGCTCAGCTTCCGCGTCGTCGGCGGGGAGCA 60

TA_1AS GCTCGAGCAGCTGGATGACGACGAGCATATCCTCAGCTTCCGCGTCGTCGGCGGCGAGCA 60

*** ** * * * ** ****** * * ** ***** *****

TA_3B_A CCGCCTCGCCAATTACCGGTCCGTGACCACCGTGAACGAGGTGGCGTCGACGGTGGCGGC 111

TA_2DS_FL CCGGCTCCGCAACTACCGGTCGGTGACCACGGTGCACCCGGCGCCGGGG---GAG----- 112

TA_3AS CCGCCTCCGCAACTACCGCTCCGTCACCTCCGTCACCGAGTTCACGGAC---CAGCCTTC 117

TA_CDM82373 CCGCCTCCGCAACTACCGCTCCGTCACCTCCGTCACCGAGTTCACGGAC---CAGCCTTC 117

TA_3B_B CCGCCTCCGCAACTACCGCTCCGTCACCTCCGTCACCGAGTTCACGGAC---CAGCCTTC 117

VIGS_TA_4AS_A CCGGCTCAAGAACTACCTCTCCGTCACCACCGTGCACCCATCCCCGGCC---GCGCCGTC 117

TA_1AS CCGCCTCCGCAACTACCGCTCCGTCACCTCCGTCACCGAGTTCGCGGAC---GAGCCTTC 117

*** *** ** **** ** ** *** * ** * ** *

TA_3B_A CGGGGCGC-CGCGGGTGACTCTGGTGGTCGAGTCGTACGTGGTGGACGTGCCGCCTGGGA 170

TA_2DS_FL -AGCG----CGTCGGCGACGCTGGTGGTGGAGTCGTACGTGGTGGACGTGCCCCCCGGGA 167

TA_3AS AGGCCCGCCCTACTGCGT----CGTTGTCGAGTCCTACGTCGTCGACGTACCGGAGGGCA 173

TA_CDM82373 AGGCCCGTCCTACTGCGT----CGTTGTCGAGTCCTACGTCGTCGACGTACCGGAGGGCA 173

TA_3B_B AGGCCCGTCCTACTGCGT----CGTTGTCGAGTCCTACGTCGTCGACGTACCGGAGGGCA 173

VIGS_TA_4AS_A CAGCGC------CACCGT----CGTCGTGGAGTCGTACGTCGTGGACGTGCCCGCGGGCA 167

TA_1AS AGGCCCGTCCTACTGCGT----CGTCGTCGAGTCCTACGTCGTCGACGTACCGGAGGGCA 173

* * ** ** ***** ***** ** ***** ** ** *

TA_3B_A ACACCAGCGACGAGACGCGCCTGTTCGTGGACACCATCGTGCGGTGCAAC 220

TA_2DS_FL ACACGCCCGAGGACACCCGCGTCTTCGTGGACACCATCGTCAAGTGCAAC 217

TA_3AS ACACCGAGGAGGACACCCGCATGTTCACCGACACCGTGGTCAAGCTCAAC 223

TA_CDM82373 ACACCGAGGAGGACACCCGCATGTTCACCGACACCGTGGTCAAGCTCAAC 223

TA_3B_B ACACCGAGGAGGACACCCGCATGTTCACCGACACCGTGGTCAAGCTCAAC 223

VIGS_TA_4AS_A ACACGACCGAGGACACCCGCGTGTTCATCGACACCATCGTCAAGTGCAAC 217

TA_1AS ACACCGAGGAGGACACCCGCATGTTCACCGACACCGTGGTCAAGCTCAAC 223

**** ** ** ** *** * *** ****** * ** * ****

**B**

1: TA_3B_A 100.00 78.37 68.08 67.61 67.61 71.63 69.01

2: TA_2DS_FL 78.37 100.00 71.83 71.83 71.83 82.86 70.42

3: TA_3AS 68.08 71.83 100.00 99.55 99.55 76.96 93.72

4: TA_CDM82373 67.61 71.83 99.55 100.00 100.00 76.96 94.17

5: TA_3B_B 67.61 71.83 99.55 100.00 100.00 76.96 94.17

6: VIGS_TA_4AS_A 71.63 82.86 76.96 76.96 76.96 100.00 75.58

7: TA_1AS 69.01 70.42 93.72 94.17 94.17 75.58 100.00

**Figure H. Evaluation of nucleotide polymorphisms in other more distantly related homologs of the amplified Ta_4AS_A VIGS cDNA insert.** A) cDNA alignment of the cDNA insert regions for Ta_4AS_A against all putative wheat ABA receptor cDNAs for which complete sequence was available within the VIGS insert region. B) Sequence identity matrix comparing Ta_4AS_A to the available cDNAs encoding the VIGS insert region. Alignments and identities obtained using Clustal Omega (Sievers et al., 2011). An ‘*’ (asterisk) indicates positions which have a single, fully conserved residue.
